# Factors Affecting the Development of Bovine Respiratory Disease: A Cross-Sectional Study in Beef Steers Shipped From France to Italy

**DOI:** 10.3389/fvets.2021.627894

**Published:** 2021-06-28

**Authors:** Barbara Padalino, Francesco Cirone, Martina Zappaterra, Daniele Tullio, Gigliola Ficco, Antonio Giustino, Linda Amarachi Ndiana, Annamaria Pratelli

**Affiliations:** ^1^Department of Agricultural and Food Science, University of Bologna, Bologna, Italy; ^2^Department of Veterinary Medicine, University of Bari, Bari, Italy; ^3^Azienda Sanitaria Locale della Provincia di Bari (ASL BA) – Local Health Authority Veterinary Service, Bari, Italy; ^4^Siciliani S.p.A. Industria Lavorazione Carne, Bari, Italy; ^5^Self Employed Veterinarian, Bari, Italy

**Keywords:** transport, cattle, virus, bacteria, health, welfare

## Abstract

Bovine respiratory disease (BRD) is a complex, multifactorial syndrome and one of the major welfare and economical concerns for the cattle industry. This 1-year cross-sectional study was aimed at documenting the prevalence of BRD-related pathogens and clinical signs before and after a long journey and at identifying possible predisposition factors. Male Limousine beef steers (*n* = 169) traveling from France to Italy were health checked and sampled with Deep Nasopharyngeal Swabs (DNS) at loading (T0) and 4 days after arrival (T1). Real-time quantitative PCR was used to quantify the presence of bovine viral diarrhea virus (BVDV), bovine respiratory syncytial virus (BRSV), bovine alphaherpesvirus 1 (BoHV-1), bovine coronavirus (BCoV), bovine adenovirus (BAdV), bovine parainfluenza virus 3 (BPIV-3), *Histophilus somni, Mannheimia haemolytica, Mycoplasma bovis*, and *Pasteurella multocida*. Weather conditions at departure and arrival were recorded, and the travel conditions were taken from the travel documentation. At T0, even if no animals displayed clinical signs, some of them were already positive for one or more pathogens. At T1, the number of animals displaying clinical signs and positive for BCoV, BAdV, BRSV, *H. somni, M. haemolytica, M. bovis*, and *P. multocida* increased dramatically (*p* < 0.001). Transport also significantly increased co-infection passing from 16.0% at T0 to 82.8% at T1 (*p* < 0.001). An extra stop during the journey seemed to favor BRSV, *M. haemolytica*, and *P. multocida* (*p* < 0.05). Weather conditions, in particular sudden climate changes from departure to arrival and daily temperature variance, were found to be predisposing factors for many of the pathogens. The farm of arrival also played a role for BRSV, BAdV, and *H. somni* (*p* < 0.05). BCoV increased dramatically, but no associations were found confirming that it spreads easily during transport phases. Our findings increased our understanding of factors increasing the likelihood of BRD-related pathogens shedding and can be useful to minimize the incidence of BRD and to implement animal transport regulations.

## Introduction

Throughout Europe, a population of about 119,357,517 cattle was estimated in 2018, of which 5,923,204 and 18,547,082 were registered in Italy and France, respectively (1). While in France many cattle are kept on pasture, in Italy, they are kept indoors, and every year about 600,000 heads travel from France to Italy for fattening and slaughter purposes (2). Transport is indeed part of the management for most livestock animals, including cattle, having different purposes, such as reaching slaughterhouse, moving to different farms, breeding, fairs, and medical procedures (3). However, transport procedures are known to be stressful for animals, having both short-term and prolonged effects on their health and welfare (4–6). Among transport-related health problems, respiratory diseases are the most common and severe (7).

Bovine respiratory disease (BRD) is a welfare and economic concern in the cattle industry. It affects the lower respiratory tract and is responsible for substantial economic short-term losses due to mortality and costs of treatments and long-term costs that are difficult to assess (7). The syndrome has a multifactorial etiology including infectious agents, host, and environmental factors, such as age, breed, genetic, nutrition, climate, commingling of animals from different sources, marketing, and particularly transport (8, 9). Transportation has been associated with an increased risk of BRD because it is responsible for immune system impairment that favors pathogen prevalence changes and pathogen proliferation (7, 8). Host's immune system suppression and up- and downregulation of the inflammatory responses due to transport stress allow opportunistic pathogens to invade tissues (10). The severity of BRD has furthermore been linked to stressors and primary viral infections, which have been proven to interfere with the mucociliary clearance of the upper respiratory tract and dysregulate the tracheal antimicrobial peptides of the respiratory innate defenses, ultimately enhancing the severity of a secondary bacterial infection (9, 11). A previous study speculated that considering viral infection as the starting point for BRD on which secondary opportunistic bacteria enter was a simplistic view of the pathogenesis of the disease, whereas a primary role of some pathogens rarely detected in the past and generally considered of minor importance was identified in eliciting the disease (12). The potential pathogenetic role for these minor pathogens and the high frequency with which co-infections occur make BRD a complex disease difficult to control.

The prevalence of BRD has been investigated in several studies, and it varies from about 4% to more than 80% (13, 14). In North America, cattle at risk frequently received metaphylactic antimicrobials upon arrival to the feedlot or following feedlot placement, to both mitigate BRD and reduce colonization and proliferation of bacterial pathogens (7). However, considering the need of reducing the use of antibiotics in livestock, it seems to be essential to investigate the factors that favor the development of BRD, and it is essential to minimize them. Preloading handling, journey duration, stocking density, deck levels, season, and environmental factors in the vehicles have all been identified as risk factors for BRD in cattle (15). However, how to transport cattle is still a matter of debate, and regulations on live animal transportation are different among countries and often not respected. For instance, a recent study reported that 437 out of the 979 controlled vehicles transporting cattle across Europe from 2009 to 2013 were found to be not in compliance with the EC 1/2005 during on-road inspections in Italy (16). In Europe, the maximum duration for cattle is set at 29 h, but in recent suggestions for the implementation of the EU 1/2005, the need to reduce the maximum duration was highlighted, and the transport of animals over long journeys should be limited as far as possible (17).

The hypothesis of the study was that a long journey (more than 20 h) from France to Italy would increase the presence of BRD-related viruses and bacteria in the Deep Nasopharyngeal Swabs (DNS), and that the latter would be associated with clinical signs, travel, and weather conditions. The objective of this cross-sectional study was to document the prevalence of the multiple pathogens related to BRD before and after traveling from France to Italy in beef steers over a 1-year time. Potential associations between the different travel conditions, weather, presence of the virus/bacteria, and clinical signs were also investigated.

## Materials and Methods

The experimental procedures were approved by the Ethics Committee of the Department of Veterinary Medicine of the University of Bari, Italy (authorization no. 16/18).

### Experimental Design and Sampling

Each year ~2,000 male beef steers travel from France to the four farms considered in the present study located in Bari Province (data of the co-author GF). These male beef steers are shipped once a week or fortnightly for fattening purposes. A power analysis was conducted using Statulator® (18) to determine the sample size to include in a 1-year cross-sectional study for a target population estimated at 2,000 animals. The number of animals to be assessed was estimated assuming an expected proportion of BRD of 14–15%, with 5% absolute precision and 95% confidence interval (CI). The used proportion of BRD-positive animals was calculated by averaging the apparent BRD prevalences of 8.8 and 21.2% as reported by Timsit et al. (13). It was therefore calculated a sample size of 170–179 animals, taking randomly only few animals (4–7) per vehicle, considering a possible number of vehicles varying from 24 to 48 traveling each year.

The experiment took place during 1 year (from February 22, 2019 to February 21, 2020). In that period, a population of 1,957 male Limousine beef steers traveled from France to the four farms monitored. Out of this total main population, a subpopulation of 1,045 animals traveling in 34 vehicles on a total of 22 different dates was included in the study. The vehicles transported from 20 to 60 animals, and 5 was the median number of animals (ranging from 3 to 6) randomly chosen among the animals traveling each time. Consequently, samples were collected in total from 169 beef steers before loading (T0) and 4 days after arrival (T1). The beef steers were 10–14 months old with a body weight of about 380 ± 20 kg, with Body Condition Scores of 3 (167/169, 98.8%) or 4 (2/169, 1.2%) (19). They originated from two assembly centers (ACs) located in France: Celmar Siret 31449234900019 (GPS location: 46.222870, 1.497133 DD; Malonze, 23300 La Souterraine, France) (AC1) and Union Altitude Siret 32313877600279 (GPS location: 45.317993, 1.764769 DD; Le Foirail, 19460 Naves) (AC2). In France, before collection at the ACs, animals were reared at pasture on different farms, located close to the ACs (i.e., <200 km). Those farms were officially free of tuberculosis and bovine leucosis. All animals were vaccinated against bluetongue serotype 8 (Merial BTV 8) in conformity with Annex III.A 85 to Regulation (EC) No. 1266/2007. At the ACs, they were grouped respecting the original penning, kept for <6 days, treated with insecticide/repellant (Cydectin® Pour-On for Cattle and Red Deer; Virbac, Carros, France), in conformity with Article 9 of Regulation (EC) No. 1266/2007, and then shipped to Italy.

At T0, official government veterinarians checked the health of each animal, and steers with clinical signs were not loaded onto the trucks. Meanwhile, at T0, two DNS (one swab per nasal cavity) were collected from each of the 169 animals. Before sampling, the nostril was wiped clean with 70% ethanol. Sterile dry swabs 13 cm long were used for sampling, and after collection, DNS were immediately stored on ice. The DNS were transported in refrigerated boxes kept in the cabin of the vehicles where the tested animals were transported. After DNS collection, animals were marked, loaded into the vehicles, and transported to the farm of destination. The beef steers considered for the present study were transported in 34 vehicles throughout all 4 seasons: 9 vehicles traveling on 4 different dates in autumn, 10 vehicles traveling on 7 different dates in winter, 4 vehicles on 4 dates in spring, and 11 vehicles traveling on 7 different dates in summer. The vehicles were 4 × 2 cab over engine prime movers (Scania®, Europe) with a three-axle enclosed flat deck semitrailer configured with two decks (Supplementary Figure 1). The vehicles had the certificate of approval according to Article 18 of the EC Regulation No. 1/2005, and belonged to a single transporter, authorized for all journeys including long journeys, according to the same EC Regulation. The vehicles were therefore equipped with suitable drinking systems, temperature sensors, and mechanical ventilation systems (i.e., 28 automatic fans at each site). During the travel, steers received water and were fed at regular intervals, in compliance with the EC Regulation 1/2005. All journeys were performed on a similar route of about 1,700 km, and they were completed in no <20 h and no more than 29 h, as reported in the intended duration on the Trade Control and Expert System (TRACES; URL: https://webgate.ec.europa.eu/sanco/traces/). For this type of journey, based on the EC 1/2005, a stop of 1 h, without unloading the animals, after the first 14 h of the journey is mandatory for permitting animals being watered and, if necessary, fed. However, some vehicles performed an extra stop for resting the animals and other logistic reasons (i.e., change of drivers), and those stops were reported in the TRACES. None of the vehicles included in this study stopped at a control post or took more than 29 h.

The animals were transported to four different farms (F1, 2, 3, 4) in Bari (Southern Italy) of the same breeder, of which two farms (F1 and F2) received the majority of the transported animals. For this reason, F3 and F4 farms were combined in a new category named “others.” Upon arrival, the marked animals were separated from the others traveling together and were kept isolated, far from the others already reared at the arrival farm. So, they were located separately in a different pen and fed with alfalfa barley silage mixed diets similar to the ones at the ACs and during the journey. None of the cattle involved was administered antimicrobials or vaccines before or during the study. None of the sampled animals died during the study.

At T1, the marked animals were clinically examined. The presence of the following clinical signs was noted down (present/absent): nasal discharge, lacrimal discharge, coughing, diarrhea, depression, polypnea, lameness, injury, and pain. For the latter three, the type of injury, the score of lameness, and pain were also noted down. Soon after the clinical examinations, two further DNS (one swab per nasal cavity) were collected from each animal, with the same procedures and precautions taken for sample collection at departure. Immediately after collection, all DNS samples were stored on ice and transported to the laboratory of Infectious Diseases of the Department of Veterinary Medicine of Bari (Italy). All the samples per truck were processed within 24 h after the collection of T1 samples.

The epidemiological status of the tested herds was not evaluated, but the animals included in the study were located separately as soon as they arrived at the farm, without any possibility of mixing. However, F1 and F2 were indirectly monitored because, during the study period, veterinarians collected DNS and ocular samples from symptomatic cattle that were tested in the same laboratory for bovine viral diarrhea virus (BVDV), bovine alphaherpesvirus 1 (BoHV-1), bovine coronavirus (BCoV), *Pasteurella multocida*, and *Histophilus somni*. In particular, on F1, two outbreaks of ocular BoHV-1 and *P. multocida* infections were detected in December 2019 and March 2019, respectively. On F2, *P. multocida* and *H. somni* were instead identified in an outbreak that developed in December 2019.

### Real-Time Quantitative PCR

Real-time quantitative PCR (RT-qPCR) was used to quantify the presence in the collected DNS of BVDV, BoHV-1, BCoV, bovine respiratory syncytial virus (BRSV), bovine adenovirus (BAdV), bovine parainfluenza virus 3 (BPIV-3), *H*. *somni, P. multocida, Mannheimia haemolytica*, and *Mycoplasma bovis*.

Dry DNS were suspended in tissue-cultured media (10% p/v), and an aliquot was employed for biomolecular analysis. Nucleic acids were extracted using the commercial kit QIAamp® cador® Pathogen Mini Kit (Qiagen GmbH, Hilden, Germany), according to the manufacturer's instructions, with an elution volume of 100 μl. The extracted samples were stored at −80°C until tested by RT-qPCR. The samples were subjected to reverse transcription in a 10 μl total volume, using random hexamers and MuLV reverse transcriptase, according to the manufacturer's protocol (GeneAmp® RNA PCR; Applied Biosystems, Applera Italia, Monza, Italy). Primers and TaqMan probe for RT-qPCR assay were used as previously described (20, 21), in the same reaction conditions, including reaction mix component and thermal cycling with an annealing temperature of 56°C (Supplementary Table 1). Briefly, 10 μl of cDNA or extracted DNA was added to the 15 μl reaction master mix (IQ™ Supermix; Bio-Rad Laboratories Srl, Milan, Italy) containing 0.6 μM of each primer and 0.4 μM probe. Thermal cycling consisted of the activation of iTaq DNA polymerase at 95°C for 10 min and 45 cycles of denaturation at 95°C for 10 s and annealing–extension at 56°C for 30 s. RT-qPCR was performed in an i-Cycler iQTM Real-Time Detection System (Bio-Rad Laboratories Srl), and the data were analyzed with the appropriate sequence detector software (version 3.0). Fluorescence was monitored during the whole RT-qPCR process. A DNS was considered positive if the fluorescence peaked between 7 and 45 cycles, following the settings reported by Kishimoto et al. (21).

### Statistical Analysis

#### Predictive and Outcome Variables

The weather parameters (average, minimal and maximal temperatures, humidity, wind speed, and quantity of precipitation) at the cities of departure and arrival were taken from a weather website (meteoblue.com). Diurnal temperature variation on the day of arrival and the changes in temperature and humidity between arrival and departure (delta, ΔT, and ΔH, respectively) were calculated as reported in Table 1.

**Table 1:** Weather and transport conditions considered as predictive variables, with their definitions and categories, during a 1-year cross-sectional study in beef steers shipped from France to Italy.

**Name**	**Definition**	**Categories**
**Weather conditions**		
Season	Season in which the tested animals were transported from France to Italy	Autumn, spring, summer, winter
Arrival temperature (AT)	The average temperature recorded in the weather website for the farm of arrival area on the day of arrival	1 (from 5 to 9°C), 2 (from 10 to 13°C), 3 (from 14 to 19°C), 4 (from 20 to 23°C), 5 (from 24 to 30°C)
Arrival humidity (AH)	The average relative humidity recorded in the weather website for the farm of arrival area on the day of arrival	Medium–low (from 60 to 70%), medium–high (from 71 to 80%), very high (from 81 to 100%)
Diurnal temperature variation	This range was calculated as the difference between the maximum and the minimum temperature recorded on the day of arrival	1 (from 0 to 5°C), 2 (from 6 to 8°C), 3 (from 9 to 11°C), 4 (from 12 to 17°C)
Delta temperature between arrival and departure (ΔT)	The difference between the average temperature recorded in the weather website for the farm of arrival area on the day of arrival and the average temperature recorded for the assembly point area on the day of departure	Low difference (from −1 to +3°C), medium difference (from +4 to +6°C), high difference (from +7 to +10°C)
Delta humidity between arrival and departure (ΔH)	The difference between the average relative humidity recorded in the weather website for the farm of arrival area on arrival and the average relative humidity recorded for the assembly point area on the day of departure	1 (from −35 to −10%), 2 (from −5 to +10%), 3 (from +15 to +30%)
Arrival wind speed	The average wind speed recorded in the weather website for the farm of arrival area on the day of arrival	Low (from 0 to 10 km/h), medium–low (from 11 to 14 km/h), medium (from 15 to 19 km/h), strong (from 20 to 24 km/h), very strong (≥25 km/h)
Arrival precipitations	The presence/absence of atmospheric precipitations recorded in the weather website for the farm of arrival area on the day of arrival	Yes, no
**Travel conditions**		
Assembly center (AC)	Center where animals from different farms converge before being loaded for transport. The animals in this study come from two assembly centers located in La Souterraine (France) and Naves (France)	AC1, AC2
Vehicles	The vehicles transporting the beef steers from the assembly centers (ACs) to the arrival farms (F)	34 vehicles
Stocking density	Stocking density during transport calculated by splitting the total kg loaded for the space available in each vehicle	Low (from 104.8 to 182.5 kg/m^2^), high (from 323.5 to 390.6 kg/m^2^)
Extra stop	The presence/absence of an extra stop	Yes, no
Farm (F)	Farm of arrival in Bari, Southern Italy	F1, F2, others (F3 + F4)
Positivity for a pathogen at T0	For each animal, the positivity of the DNS for a certain pathogen at T0	Presence (1), absence (0)

The data related to the vehicle (number of animals traveling, total kg loaded, available space), estimated journey duration, and presence of a resting stop(s) during transport were taken from the TRACES. The estimated journey duration varied from 20 to 28 h depending on the presence of an extra stop. Unfortunately, it was not possible to record the effective journey since many of the vehicles arrived at night at the destination farm and we were not allowed to have the journey log. The estimated journey duration was not considered as a predictive variable for avoiding confounding factors since it was collinear with the presence of more than a resting stop. Stocking density was calculated by splitting the total kg loaded for the space available in each vehicle.

Weather and travel conditions were clustered in categories and thus considered as predictive variables in the regression analysis (Table 1). The positivity for each pathogen (positive/negative, 1/0) of the DNS collected from the beef steers at T0 was also used as predictive variable for the regression analyses performed for the positivity for the same pathogen at T1.

For each animal, the total number of positivities to the tested pathogens was calculated at T0 and T1. Depending on the number of pathogens identified in each animal, the following categories were created for both T0 and T1: negative, single positive, and positive for 2, 3, 4, 5, and 6 pathogens. Based on those data, a category named “co-infection” was created with two values (yes/no): (i) animals positive for at least two pathogens (yes) and (ii) animals negative or single positive (no).

#### Descriptive Statistics

Descriptive statistics were performed on the weather conditions detected for the 44 sampling dates, with the minimum, maximum, and average values obtained with Microsoft Office Excel. Descriptive statistics of all predictive variables, identified as categorical, were performed using the Statulator® online free software and reported as counts and percentages. The number of animals with clinical signs (presence/absence), positive for the pathogens (positive/negative), and the co-infection category (yes/no) were counted and then compared between T0 and T1 with a two-tailed McNemar's χ^2^ test statistics using the online tool of the Center for Clinical Research Biostatistics (CCRB; URL: https://www2.ccrb.cuhk.edu.hk/stat/confidence%20interval/McNemar%20Test.htm). The clinical signs and pathogens that resulted to be significant with the McNemar's χ^2^ test and had at least 10 positive observations at T1 (10/169, a prevalence of more than 5%) (22) were further investigated in the subsequent statistical analyses.

#### Regression Analysis

Collinearity between categorical variables was tested using Kendall's tau statistic with the *cor.test* function in R environment (23). Categories 4 and 5 of the arrival temperature (AT) variable were collinear with the summer season. Each clinical sign (presence/absence, 1/0) and each pathogen (positive/negative, 1/0) at T1 were used as dependent categorical dichotomous variables (dummy variables) in univariate logistic regression models, in order to identify the predictive variables to be included in the stepwise backward selection and find the final multivariable model for each clinical sign and pathogen. Additionally, only for the dummy dependent variables of the positivity to bacteria at T1, the positivity to the viruses at T1 (positive/negative, 1/0) was used as a predictive variable. No associations were noticed between the AC variable and the dependent variables of the presence of clinical signs and pathogens at T1, nor between the 34 vehicles and the dependent variables of the presence of clinical signs and pathogens at T1. Thus, ACs and vehicles were not further considered in the subsequent statistical analyses. Results of the univariate logistic regression are reported as odds ratio (OR), 95% CI, and *p*-values. *p*-values were calculated using the Wald test, and for each outcome, the variables that showed a *p* <0.25 were considered for inclusion in the stepwise multiple regression. Due to the collinearity between categories 4 and 5 of the AT and the summer season, if season and AT were both significant in univariate logistic regression, only the AT was further considered for the inclusion in the stepwise multiple regression. A stepwise backward elimination procedure was indeed conducted for each dependent variable (presence/absence of clinical signs and positivity/negativity to the pathogens) to test the combined effect of the predictive variables. The predictive variables were removed until all variables in the final model had a *p* < 0.10 (24). The final multivariable models resulting from the stepwise backwards selection are presented as OR, 95% CI, and *p*-value. The scripts used to perform the univariate logistic and stepwise multiple regressions were a combination of functions in the packages *nlme, lsmeans, lme4*, and *car* in R environment (23).

Univariate logistic regression was also used to test the associations between the presence/absence of clinical signs (dependent variable) and the dummy variable of the positivity to the pathogens (predictive variable).

#### Multiple Correspondence Analysis

Multiple correspondence analysis (MCA) was performed to detect underlying structures in the dataset, using the dummy variables of the clinical signs and the positivity to the pathogens, and the predictive variables defined in Table 1. The results are represented by the proportion (in %) of total variance (called “inertia” in MCA) explained by each dimension (Dim), the list of the contributions of the variable categories to each Dim (the highest the contribution of a category to a Dim, the most important it is in explaining the inertia of the dataset), and the coordinates of the categories in each Dim. The absolute value of the coordinates allows identifying the weight of each category in a Dim, and the sign of the coordinates provides also an indication concerning the correlation existing among the categories in each Dim. The MCA was performed using the *FactoMineR* package, and the results were plotted using the packages *factoextra, gplots*, and *grDevices* in R environment (23).

## Results

### Descriptive Statistics

The weather conditions recorded on the 44 sampling dates (22 on departure and 22 on arrival) are summarized in Supplementary Table 2. The average temperatures noted over the different seasons were generally comparable between the ACs at T0 and the arrival farms at T1, with the average temperatures ranging from 9°C in winter to 22°C in summer at T0 and from 10°C in winter to 24°C in summer at T1. However, the minimum temperatures observed at T1 were on average higher than those observed at T0. In particular, the sampling dates in autumn and spring showed 7°C and 6°C as minimum temperatures, respectively, whereas the minimum temperatures noticed in the same two seasons were near to 0 at T0 (2°C in autumn and 1°C in spring). The greater diurnal temperature variation was observed at T0 during the summer period when the observed minimum temperature was 12°C and the maximum temperature was 35°C. During spring and summer, the average and maximum humidity at T1 were overall higher than those at T0 (in summer 59–80% at T0 vs. 73–80% at T1, in spring 60–75% at T0 vs. 78–85% at T1), whereas in autumn and winter, the humidity percentages were higher at T0 than at T1 (in autumn 82–100% at T0 vs. 73–85% at T1, in winter 78–85% at T0 vs. 76–80% at T1). Average and maximum atmospheric precipitations were notably higher in autumn, winter, and spring at T0 (6–14 mm in autumn, 4–12 mm in winter, 4–16 mm in spring) than at T1 (1–2 mm in autumn, 1–8 mm in winter, 1–2 mm in spring).

Overall, AC1 and AC2 were equally represented in the sample, with a similar number of steers coming from each AC (78/169, 46.1% from AC1; 91/169, 53.9% from AC2). Most of the transported animals (155/169, 91.7%) were headed to two (F1 and F2) out of the four farms. About half of the steers (74/169, 43.8%) traveled at lower stocking densities (from 104.8 to 182.5 kg/m^2^), whereas the remaining ones (95/169, 56.2%) were transported at higher stocking densities (from 323.5 to 390.6 kg/m^2^). The transport distance was comparable among the journeys (about 1,700 km); however, 30.2% (51/169) of the vehicles performed an extra stop during the journey for logistic reasons (Table 2). Descriptive statistics of the explanatory variables related to the weather conditions are shown in Supplementary Table 3.

**Table 2:** Frequency table of beef steers (*n* = 169) transported from France to Italy for the variables related to travel conditions and co-infection at T0 (departure) and T1 (4 days after arrival).

**Variable**	**Count**	**%**
Assembly center (AC)		
AC1	78	46.1
AC2	91	53.9
Total	169	100.0
Farm (F)		
F1	74	43.8
F2	81	47.9
Others (F3 + F4)	14	8.3
Total	169	100.0
Stocking density		
Low (from 104.8 to 182.5 kg/m^2^)	74	43.8
High (from 323.5 to 390.6 kg/m^2^)	95	56.2
Total	169	100.0
Extra stop		
No	118	69.8
Yes	51	30.2
Total	169	100.0
Number of animals positive to pathogens at T0		
Negative	90	53.2
Single positive	52	30.8
Positive for 2 pathogens	16	9.5
Positive for 3 pathogens	8	4.7
Positive for 4 pathogens	2	1.2
Positive for 5 pathogens	1	0.6
Total	169	100.0
Number of animals positive to pathogens at T1		
Negative	4	2.4
Single positive	25	14.8
Positive for 2 pathogens	49	29.0
Positive for 3 pathogens	42	24.8
Positive for 4 pathogens	35	20.7
Positive for 5 pathogens	13	7.7
Positive for 6 pathogens	1	0.6
Total	169	100.0

### Travel Effect on the Prevalence of Clinical Signs and Pathogens at T1

As reported in Table 3, only healthy animals that showed no clinical signs of coughing, depression, diarrhea, injury, lacrimal discharge, lameness, nasal discharge, pain, or polypnea were transported. Despite no clinical signs at T0, some of the DNS were positive. In particular, 48 beef steers (28.4%) were positive for *H. somni*, 21 to *P. multocida* (12.4%), 14 to *M. haemolytica* (8.3%), 7 to *M. bovis* (4.1%), and 4 to BAdV (2.4%).

**Table 3:** Beef steers (*n* = 169) traveling from France to Italy.

	**T0**		**T1**		**McNemar's** **χ**^**2**^ **test**	
	**N**	**%**	**N**	**%**	**Statistics**	***p*-value**
Injury	0/169	0.0	1/169	0.6	n.e.	n.e.
Pain	0/169	0.0	1/169	0.6	n.e.	n.e.
Depression	0/169	0.0	1/169	0.6	n.e.	n.e.
Polypnea	0/169	0.0	1/169	0.6	n.e.	n.e.
Coughing	0/169	0.0	20/169	11.8	18.05	<0.001
Diarrhea	0/169	0.0	28/169	16.6	26.04	<0.001
Lacrimal discharge	0/169	0.0	33/169	19.5	31.03	<0.001
Nasal discharge	0/169	0.0	90/169	53.3	88.01	<0.001
BoHV-1	0/169	0.0	0/169	0.0	n.e.	n.e.
BVDV	0/169	0.0	0/169	0.0	n.e.	n.e.
BRSV	1/169	0.6	15/169	8.9	12.07	<0.001
BCoV	27/169	16.0	110/169	65.1	67.91	<0.001
BPIV-3	0/169	0.0	4/169	2.4	2.25	0.133
BAdV	3/169	1.84	18/169	10.7	11.53	<0.001
*H. somni*	48/169	28.4	148/169	87.6	92.46	<0.001
*M. haemolytica*	14/169	8.3	27/169	16.0	4.36	0.036
*M. bovis*	7/169	4.1	78/169	46.1	65.33	<0.001
*P. multocida*	21/169	12.4	60/169	35.5	29.47	<0.001
Co-infection	27/169	16.0	140/169	82.8	101.98	<0.001

*Descriptive statistics and comparison between departure (T0) and 4 days after arrival (T1) of the observed prevalence of the animals displaying clinical signs and positivity for the investigated nasopharyngeal pathogens.*

*n.e., not estimable*.

At T1, while the number of animals showing injury, pain, depression, lameness, or polypnea was low (1/169, 0.6%), the number of animals displaying coughing (20/169, 11.8%), diarrhea (28/169, 16.6%), and lacrimal (33/169, 19.5%) and nasal (90/169, 53.3%) discharges increased dramatically (*p* < 0.001). The number of positive DNS also increased significantly for almost all the investigated pathogens, except for BPIV-3, with only four animals positive at T1 (4/169, 2.4%; *p* > 0.050), and for BVDV and BoHV-1, which were always negative both at T0 and T1. Transport also significantly increased co-infection, which went from 16.0% at T0 to 82.8% at T1 (*p* < 0.001).

### Factors Associated With Clinical Signs at T1

The complete list of Wald test *p*-values calculated between the predictive variables and the presence of clinical signs at T1 is reported in Supplementary Table 4. The OR, 95% CI, and *p*-values of the variables significantly associated with the presence of clinical signs at T1 in the univariate logistic regressions are reported in Supplementary Table 5.

Table 4 reports the OR, 95% CI, and *p*-values of the variables retained in the final multiple regression models. The presence of coughing was associated with stocking density (*p* = 0.004), ΔT (*p* = 0.002), and arrival precipitations (*p* < 0.001). In particular, the odds of coughing were increased 10-fold in animals transported at high stocking density, by 11 times when ΔT was of 4–6°C, and by 35 times when there were no arrival precipitations.

**Table 4:** Final multivariable regression models for the dummy dependent variables of the presence/absence of clinical signs.

**Variable**	**Category**	**Final multivariable model**		
		**OR**	**95% CI**	***p***
**Dependent variable: coughing**				
Stocking density	Low	Ref		
	High	10.33	2.88–46.86	<0.001
ΔT	Low	Ref		
	Medium	11.62	3.54–44.89	<0.001
	High	n.e.	n.e.	0.991
Arrival precipitations	Yes	Ref		
	No	35.38	5.65–707.05	0.001
**Dependent variable: diarrhea**				
ΔT	Low	Ref		
	Medium	2.30	0.76–7.06	0.136
	High	60.37	7.43–1,342.21	<0.001
ΔH	Medium	Ref		
	Low	0.45	0.02–3.98	0.516
	High	5.84	1.74–26.86	<0.001
**Dependent variable: lacrimal discharge**				
F	F1	Ref		
	F2	24.97	6.29–170.90	<0.001
	Others	n.e.	n.e.	0.992
ΔH	1 (−35 to −10%)	ref		
	2 (−5 to +10%)	4.04	0.68–77.56	0.202
	3 (+15 to +30%)	19.98	3.29–389.81	0.007
**Dependent variable: nasal discharge**				
F	F1	Ref		
	F2	8.03	2.10–40.71	0.005
	Others	3.13	0.82–13.30	0.103
AT	1 (5–9°C)	Ref		
	2 (10–13°C)	13.22	2.72–84.56	0.003
	3 (14–19°C)	140.65	20.32–1,250.12	<0.001
	4 (20–23°C)	38.05	5.04–361.04	<0.001
	5 (24–30°C)	65.77	12.40–448.29	<0.001

*The symptoms were observed 4 days after arrival in 169 beef steers transported from France to Italy. Data are presented as odds ratio (OR), 95% confidence interval (95% CI), and p-value (p).*

*n.e., not estimable; Ref, reference; AT, arrival temperature; ΔT, delta temperature between arrival and departure; ΔH, delta humidity between arrival and departure; F, farm of arrival*.

Diarrhea was mainly related to ΔT (*p* < 0.001) and ΔH (*p* < 0.001). In particular, high difference category for ΔT (from +7 to +10°C) and ΔH (from +15 to +30%) increased, respectively, by 60 and almost 6 times the presence of diarrhea compared with lower Δ.

Lacrimal discharge was related to the farm of arrival (*p* < 0.001) and to ΔH (*p* = 0.022). Animals transported to F2 were almost 25 times more likely to display lacrimal discharge than beef steers in F1, and ΔH (between +15 and +30% than T0) was associated with a 20-time increase in the likelihood of showing lacrimal discharge compared with the ΔH from −35 to −10%.

Nasal discharge was associated with the farm of arrival (*p* < 0.001) and AT (*p* < 0.001). The animals were eight times more likely to display nasal discharge if transported to F2 than to F1, and if AT was above 10°C.

### Factors Associated With Viral Infections at T1

The complete list of Wald test *p*-values calculated between the predictive variables and the viral infections at T1 is reported in Supplementary Table 6. The OR, 95% CI, and *p*-values of the variables significantly associated with the presence of viral infections at T1 in the univariate logistic regressions are reported in Supplementary Table 7.

Table 5 reports the OR, 95% CI, and *p*-values of the variables retained in the final multiple regression models. BRSV was found to be associated with the farm of arrival (*p* = 0.029), the presence of an extra stop during the transport (*p* = 0.020), and the AH (*p* < 0.001). For the variable farm of arrival, the category “others” was more than 20 times more likely to have beef steers positive for BRSV than F1, and this possibility was even higher if at arrival there was a medium–low AH than a medium–high AH.

**Table 5:** Final multivariable regression models for the dummy dependent variables of positivity/negativity to viral infections.

**Variable**	**Category**	**Final multivariable model**		
		**OR**	**95% CI**	***p***
**Dependent variable: BRSV positivity**				
F	F2	Ref		
	F1	2.39	0.48–17.68	0.321
	Others	23.08	2.14–624.09	0.021
Extra stop	No	Ref		
	Yes	5.52	1.30–28.99	0.026
AH	Medium–high	Ref		
	Medium–low	37.03	4.70–1,163.15	0.006
	Very high	n.e.	n.e.	0.995
**Dependent variable: BAdV positivity**				
F	F1	Ref		
	F2	48.82	6.33–1,167.10	0.002
	Others	7.79	0.88–174.69	0.097
Stocking density	High	Ref		
	Low	23.60	4.99–179.78	<0.001
ΔH	1 (−35 to −10%)	Ref		
	2 (−5 to +10%)	4.81	0.58–103.48	0.193
	3 (+15 to +30%)	49.46	5.98–1,227.89	0.002

*The positivities were observed 4 days after arrival in the DNS from 169 beef steers transported from France to Italy. Data are presented as odds ratio (OR), 95% confidence interval (95% CI), and p-value.*

*n.e., not estimable; Ref, reference; F, farm of arrival; AH, arrival humidity; ΔH, delta humidity between arrival and departure*.

BAdV was found to associated with the farm of arrival (*p* = 0.007), the stocking density (*p* = 0.002), and the ΔH (*p* = 0.006). The odds of having beef steers positive to BAdV at T1 were 48 times higher if transported to F2, almost 50 times with a ΔH of category 3 (+15 to +30%), and 20 times higher if transported at low stocking densities.

On the other hand, BCoV did not show any significant associations with the investigated factors.

### Factors Associated With Bacterial Infections at T1

The complete list of Wald test *p*-values calculated between the predictive variables and the bacterial infections at T1 is reported in Supplementary Table 8. The OR, 95% CI, and *p*-values of the variables significantly associated with the presence of bacterial infections at T1 in the univariate logistic regressions are reported in Supplementary Table 9.

Table 6 reports the OR, 95% CI, and *p*-values of the variables retained in the final multiple regression models. The positivity to *H. somni* was associated with the farm of arrival (*p* < 0.001) and the diurnal temperature variation (*p* < 0.001). Indeed, the odds of finding animals positive to *H. somni* were 30 times higher when beef steers arrived at F1 than at F2, and there was the diurnal temperature variation category 4 (from 12 to 17°C).

**Table 6:** Final multivariable regression models for the dummy dependent variables of positivity/negativity to bacterial infections.

**Variable**	**Category**	**Final multivariable model**		
		**OR**	**95% CI**	***p***
**Dependent variable:** ***H. somni*** **positivity**				
F	F2	Ref		
	F1	36.28	5.72–775.03	0.002
	Others	n.e.	n.e.	0.992
Diurnal temperature variation	4 (12–17°C)	Ref		
	1 (0–5°C)	61.28	7.91–1,377.89	<0.001
	2 (6–8°C)	101.45	14.67–2,130.59	<0.001
	3 (9–11°C)	53.16	6.99–1,175.43	<0.001
**Dependent variable:** ***M. haemolytica*** **positivity**				
Extra stop	No	Ref		
	Yes	6.60	1.40–48.02	0.028
Diurnal temperature variation	3 (9–11°C)	Ref		
	1 (0–5°C)	29.01	4.65–312.96	0.001
	2 (6–8°C)	2.17	0.41–14.57	0.380
	4 (12–17°C)	n.e.	n.e.	0.992
Arrival precipitations	Yes	Ref		
	No	17.38	2.87–346.72	0.011
BRSV positivity at T1	No	Ref		
	Yes	9.95	1.70–74.32	0.015
**Dependent variable:** ***M. bovis*** **positivity**				
Stocking density	Low	Ref		
	High	2.39	1.06–5.60	0.038
AT	1 (5–9°C)	Ref		
	2 (10–13°C)	5.07	1.52–20.47	0.013
	3 (14–19°C)	2.19	0.42–1.22	0.356
	4 (20–23°C)	1.44	0.23–9.68	0.702
	5 (24–30°C)	1.37	0.29–6.79	0.688
**Dependent variable:** ***P. multocida*** **positivity**				
Stocking density	High	Ref		
	Low	2.76	1.26–6.14	0.028
Extra stop	No	Ref		
	Yes	3.16	1.44–6.99	0.002
*P. multocida* positivity at T0	No	Ref		
	Yes	5.62	1.90–19.40	0.002

*The positive DNS were observed 4 days after arrival (T1) in 169 beef steers transported from France to Italy. Data are presented as odds ratio (OR), 95% confidence interval (95% CI), and p-value (p).*

*n.e., not estimable; Ref, reference; F, farm of arrival; AT, arrival temperature*.

*M. haemolytica* was associated with the presence of an extra stop during transport (*p* = 0.013), the diurnal temperature variation (*p* < 0.001), and the arrival precipitations (*p* < 0.001). The odds of finding animals positive to *M. haemolytica* increased by over 6 times if an extra stop was performed during transport, 29 times with the category 1 (from 0 to 5°C) of diurnal temperature variation when compared with category 3 (from 9 to 11°C), 17 times if arrival precipitations were absent, and 10 times if the animal was also positive for BRSV.

*M. bovis* positivity was related to the AT (*p* = 0.027) and the stocking density (*p* = 0.035). The odds of finding animals positive to *M. bovis* increased by five times with category 2 (from 10 to 13°C) of AT compared with category 1 (from 5 to 9°C) and by more than two times with high stocking densities.

*P. multocida* was associated with the positivity for *P. multocida* at T0 (*p* = 0.002), the stocking density (*p* = 0.029), and an extra stop during transport (*p* = 0.002). The odds of finding animals positive to *P. multocida* increased by more than five times if they were already positive to this pathogen at T0, by three times with the presence of an extra stop during transport, and by more than two times if transported at low stocking densities.

### Association Between the Presence of Clinical Signs and Pathogens at T1

**Figure 1:**
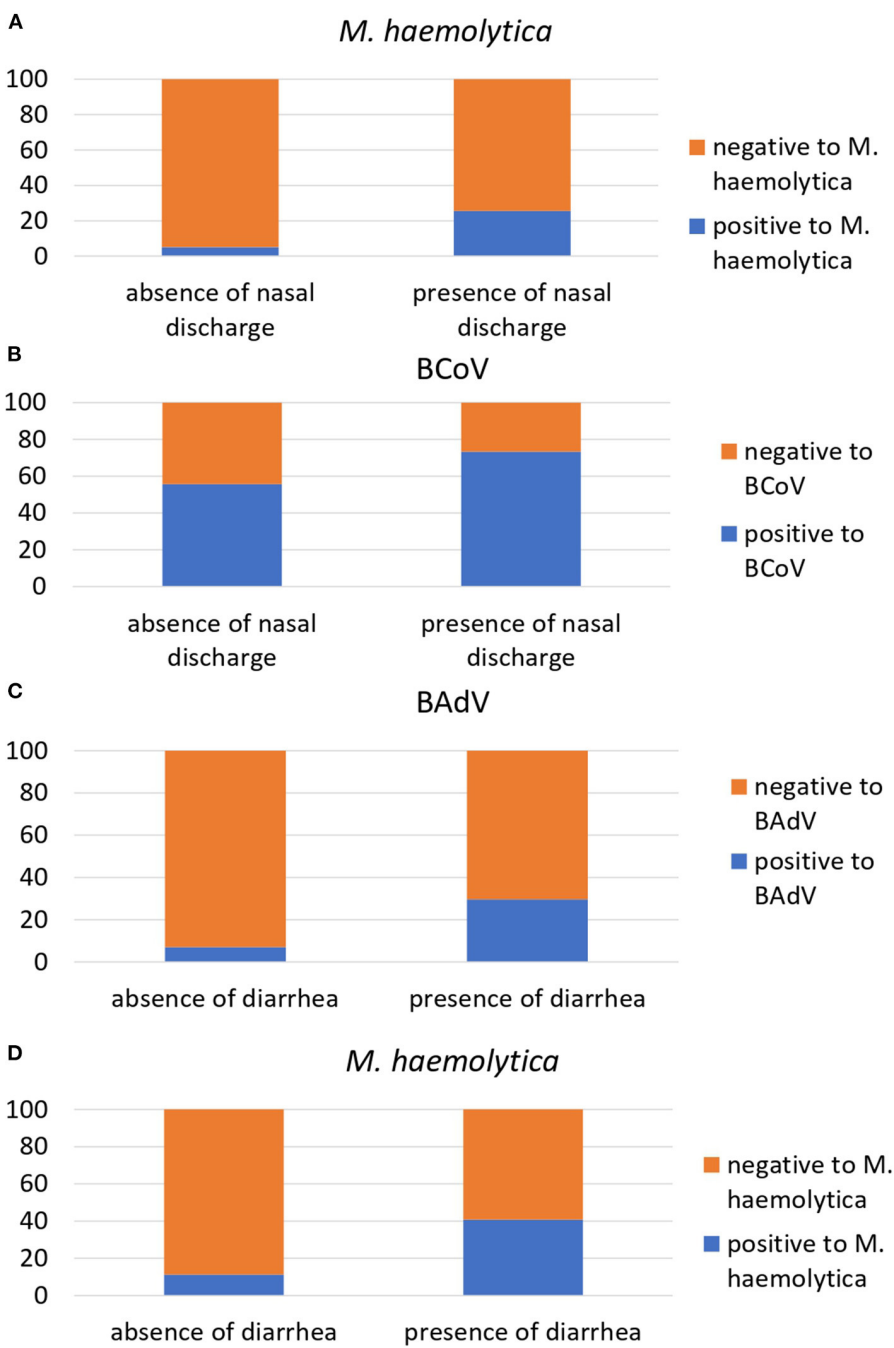
Associations between the presence/absence of nasal discharge and *M. haemolytica* at T1 **(A)**, nasal discharge and BCoV at T1 **(B)**, diarrhea and BAdV at T1 **(C)**, and between diarrhea and *M. haemolytica* at T1 **(D)** in 169 beef steers transported from France to Italy. Each table reports the relative prevalence of the pathogens in animals with or without clinical signs.

Figure 1 shows the results of the univariable regression analysis. Among the observed clinical signs, the presence of nasal discharge was associated with the positivity to *M. haemolytica*; the animals displaying nasal discharges were six times more likely to have resulted to be positive to *M. haemolytica* (OR = 6.44, 95% CI = 2.33–22.79, *p* = 0.001) and two times more likely to have resulted to be positive to BCoV (OR = 2.19, 95% CI = 1.15–4.20, *p* = 0.017). Beef steers with diarrhea were instead five times more likely to have BAdV (OR = 5.56, 95% CI = 1.91–15.92, *p* = 0.001) and *M. haemolytica* infections (OR = 5.41, 95% CI = 2.12–13.76, *p* < 0.001).

### Identification of Variables Shaping the Multivariate Structure of the Dataset

MCA identified the underlying structures in the dataset, associations and correlations linking the categorical variables, and the most important variables shaping the dataset inertia. The top 10 new dimensions identified by MCA explained 66.8% of the total inertia (Supplementary Figure 2). The two dimensions explaining the highest inertia percentages were Dimension 1 (Dim1) and Dimension 2 (Dim2), accounting for 11.6% and 10.2% of the total inertia, respectively. The contribution of the variable categories (in %) to the definition of the dimensions is reported in Supplementary Table 10, whereas the complete list of the coordinates for all the variable categories is reported in Supplementary Table 11.

**Figure 2:**
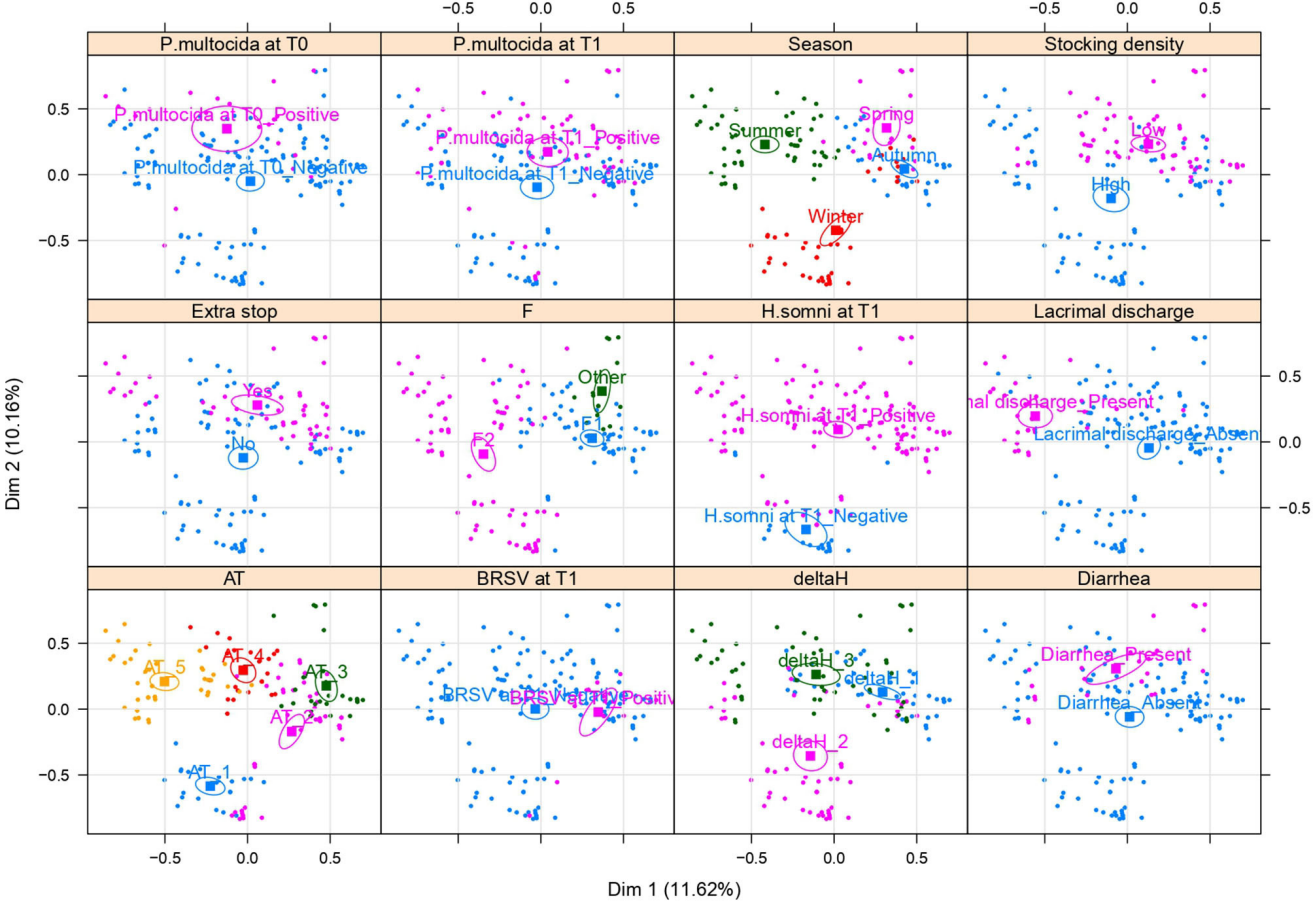
The multiple correspondence analysis (MCA) biplot with Dimension 1 (Dim1) as x-axis and Dimension 2 (Dim2) as y-axis, with the explained variance between brackets. Different colors and confidence ellipses are drawn for the main variables entering the first two dimensions and explaining most of the variance noticed in the sample of 169 beef steers transported over a long-distance journey from France to Italy. F, farm of arrival; AT, arrival temperature; ΔH, delta humidity between arrival and departure.

**Figure 3:**
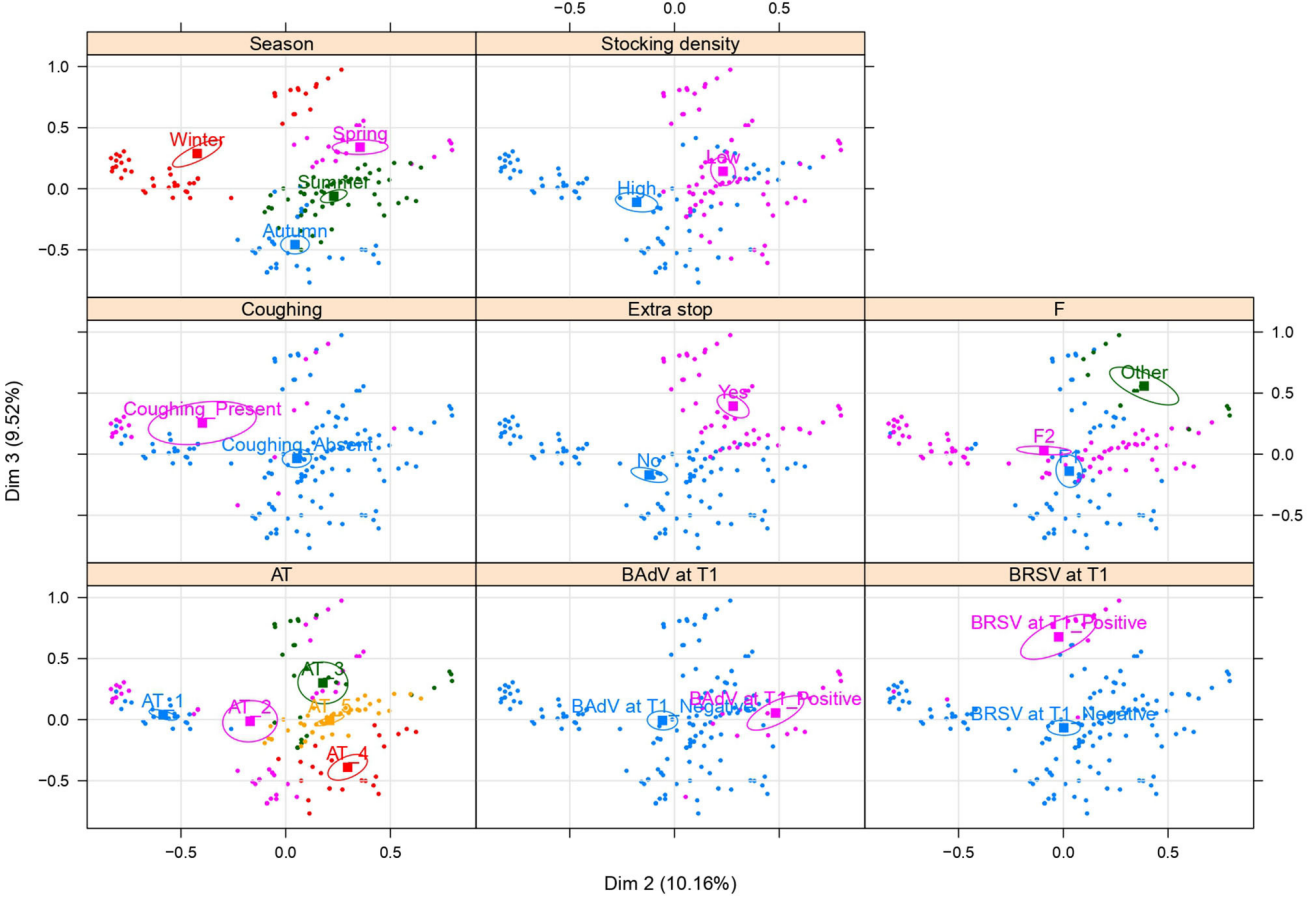
The multiple correspondence analysis (MCA) biplot with Dimension 2 (Dim2) as x-axis and Dimension 3 (Dim3) as y-axis, with the explained variance between brackets. Different colors and confidence ellipses are drawn for the main variables entering the first two dimensions and explaining most of the variance noticed in the sample of 169 beef steers transported with a long-distance journey from France to Italy. F, farm of arrival; AT, arrival temperature.

The multivariate structure of the samples is graphically represented in Figures 2, 3, where only the variables contributing the most to the inertia explained by each dimension have been reported. Figure 2 shows a reproduction of the same MCA biplot with Dim1 as x-axis and Dim2 as y-axis, with different colors and confidence ellipses for the variables showing clusters (and thus defining the two main dimensions). The variable categories contributing the most to the total inertia of the dataset structure were the season and the farm of arrival determining the subsequent clusterization visible in the biplot for the other variables. Comparing the biplots in Figure 2, it is possible to note that the animals showing lacrimal discharge at T1 were mainly those that were transported in summer to F2, where temperatures ranging from 24 to 30°C were noticed (category 5 of the AT variable). The animals that were negative to *H. somni* at T1 were those that were transported during winter to F2, where temperatures ranged from 5 to 9°C (category 1 of AT), and with a difference in the humidity comprised in category 2 (from −5% to +10%) of ΔH. The animals displaying DNS positive to BRSV were those that were transported during the winter season and at the time of arrival at F1 or “others” found temperatures comprised in category 3 (from 14 to 19°C) of the AT variable. Furthermore, it is possible to note comparing the biplots in Figure 2 that the animals positive for *P. multocida* at T1 were those already positive for this pathogen at T0, transported at low stocking densities (from 104.8 to 182.5 kg/m^2^), and with an extra stop during transport. Finally, beef steers displaying diarrhea at T1 were those subjected to high ATs (categories 4 and 5 of the AT) and an abrupt increase in humidity at arrival (category 3 of the ΔH). Figure 3 shows the same MCA biplot with Dim2 as x-axis and Dim3 as y-axis, with different colors and confidence ellipses for the variables showing clusters (and thus defining the second and the third main dimensions). Comparing the biplots in Figure 3, it is possible to note that the animals positive at T1 for BRSV were all transported at low stocking densities and during winter, whereas those positive at T1 for BAdV were transported at low stocking densities but mainly during spring and summer. It is also clear the presence of an association between winter and coughing, as beef steers transported during this season had a greater prevalence of this clinical sign.

## Discussion

This study documented the prevalence of the clinical signs and pathogens related to BRD after a long-distance journey from France to Italy in beef steers. Several associations between the different weather and travel conditions (e.g., season, diurnal temperature variation, stocking density, farms of arrival) and the presence of virus/bacteria and clinical signs recorded 4 days after arrival were identified. Our findings strongly support our hypothesis. They confirmed that the prevalence of BRD-related pathogens increased after the tested journey length and suggested that weather conditions and the presence of an extra stop during transport may also be factors increasing the likelihood of BRD and its clinical signs. To our knowledge, this is the first study on the effects of the difference of temperature and humidity between arrival and departure on the occurrence of BRD-related pathogens and clinical signs in a consistent sample of beef steers (169 animals) traveling on a similar long-distance journey throughout the whole year. Our results may be useful to minimize the incidence of BRD and to propose best practices for cattle transportation.

A valuable observation point of the sanitary conditions of cattle in Europe was provided. In Europe, there are more than 4 million cattle transported each year across the member states, with more than 117,000 cattle consignments registered in 2015 (25). Italy is one of the largest cattle importers in the EU (2), and France is Italy's main supplier of cattle for fattening and slaughter, with more than 600,000 heads imported from France to Italy in 2016 (2). The fitness for travel of the tested animals was performed by official veterinarians, but despite this, cases of positivity were registered before departure. Our results highlight therefore the role of live animal transportation as a possible way to move pathogens across Europe. However, long-distance journeys are not only a biosecurity hazard (26) but also a major welfare concern worldwide (27, 28). It is well-known that long travels are stressful events affecting the animal immune system and increasing the pathogen shedding, and ultimately result in production efficiency impairment and health problems onset (14, 29, 30). Over the last years, the number of animals transported for long-distance journeys has greatly increased, due to the increased trade in live animals between different countries and the establishment of fewer and larger growing/finishing farms and slaughter facilities often built far from the farms (31). Consequently, minimizing sufferance and poor health and welfare in the transported animals is crucial for lowering the risks of disease spread, and this is achievable only by improving the welfare standards before, during, and after a journey. Our findings are of interest not only for Europe but also for other countries whose dimensions require long journeys, such as North America or Australia.

Our results reinforce the argument that transport represents a risk factor in the development of BRD (32). The prevalence of BRD-related pathogens (BAdV, BCoV, BRSV, *H. somni, M. bovis, M. haemolytica*, and *P. multocida*) and clinical signs (coughing, diarrhea, nasal and lacrimal discharges) increased dramatically after travel, and extreme dysbiosis of the nasopharyngeal tract was observed, with a dramatic increase in co-infection (i.e., prevalence of concurrent infections increased by 500% after travel). Our results support the idea that long-distance transportation favors respiratory tract dysbiosis with concurrent bacterial and virus infections leading to BRD (14, 33–35). The drastic increase in co-infection found gets even more interesting when considering that all journeys included in this study complied with EC 1/2005, and all were within the maximum journey duration allowed. Extensive literature exists on the effect that different travel distances may have on stress-related physiological responses (36, 37) and BRD-related pathogens (15, 38). Our findings support the idea that the maximum travel time allowed should be reduced, as already pointed out in previously published activity reports to the European Parliament (17). However, the journey time represents only one of the variables affecting the occurrence of BRD, and other factors are supposed to play roles in the occurrence of this multifactorial disease (33). Consequently, analyzing individually which factors influence the prevalence of pathogens and clinical signs may provide suggestions for further implementing EC 1/2005 and provide best practices for cattle transportation.

Multiple factors affecting the occurrence of this complex disease have been investigated. BRD predisposing factors are weather conditions, including season (39–42), management factors at the ACs or sale barns (43), stocking density (44), human factors (33, 39), and age and gender of the animals (33). Our results strongly indicated that season and its associated variables, such as ΔT, ΔH, AT, AH, diurnal temperature variation, and arrival wind speed, resulted to be crucial for the prevalence of the BRD-related pathogens and clinical signs investigated in the present study. Autumn has been found as one of the possible predisposing factors in the outbreak of this disease in cattle transported across North America (39–41). However, subsequent scientific literature suggested that the association between autumn and BRD occurrence may also be caused by the greater numbers of cattle marketed during that season, with implications on higher crowding, commingling, and competition for feeding at sale barns (33). We were not able to find the same strong association between all tested BRD-related pathogens and autumn, and this could be due to the difference in climate between North America and South Europe and to the different production strategies. Finally, it is worth noting the effect of climate changes on seasons, and consequently, also based on our results, to safeguard the welfare of the cattle during the journey, it seems more appropriate to plan journeys accordingly with the weather forecasting and mitigate the weather conditions than reducing the journeys in a particular season. Overall, our results suggest that different combinations of weather and travel conditions can increase or decrease the odds of animals being positive for a particular virus or bacterium, depending also on the characteristics of the pathogens.

Several bacteria have been linked with BRD. The positivity for *M. bovis* increased 10 times after traveling. *M. bovis* has been isolated from the lungs of animals affected by BRD, and in several cases of feedlot cattle with chronic, non-responsive respiratory disease (45, 46). However, it is often regarded as an opportunist in cattle respiratory tract, whose virulence is enhanced by other concomitant infections (47). Interestingly, in the present study, the prevalence of DNS positive to *M. bovis* increased when the AT was between 10 and 13°C and animals were transported in autumn to F1, with a strong arrival wind speed and a ΔH between −35 and −10%. The positive association between *M. bovis* and autumn agrees with the literature (39–41). This could be due to temperatures that favor bacterial growth and to the fact that the animals were grazed for the summer months on pasture in France. Sharing pasture between cattle and other animal species was proven to be a risk factor in *M. bovis* shedding (48). However, the sanitary condition of the farm (people, equipment, and managing decisions) may play also a primary role in *M. bovis* shedding, as suggested also by previous research on this pathogen (49). Feedlot placement affects the nasopharyngeal microbiota of beef cattle (7), and it is not surprising that the farm of arrival was also one of the major factors affecting the prevalence of *H. somni* (14). In particular, the animals being more likely positive to this pathogen were those transported to F1, suggesting that this bacterium could have been endemic in that farm. An outbreak of *H. somni* infection was found in young male cattle from an autochthonous breed reared in a farm located in the Center of Italy during the first months of 2016 (50). Schiavon et al. (51) reported that in Italy, *H. somni*-related pathologies mainly occur in beef steers aged between 4 and 10 months during autumn and winter seasons and their onset can be affected by the stress resulting from cold and changing weather conditions (52). Our animals belonged to that age category, and we found an increase of positivity in autumn and summer, but not in winter. Despite the association between summer and *H. somni* positivity may appear surprising at first, it is worth mentioning that the tested vehicles crossed the Alps; the temperature curve during these types of journeys in summer may be extremely stressful for the transported animals, with temperatures dropping from 25 to 6°C (53) and then increasing again to 30°C during the travel. While the role of *H. somni* in BRD onset is still unclear, *P. multocida* is among the key pathogens associated with bovine pneumonia (54). In our study, about 35% of the animals resulted to be positive for *P. multocida* after the journey, and low stocking densities, pre-existing infection, and the presence of an extra stop during transport resulted to be predisposing factors. *P. multocida* transmission mainly occurs *via* direct contact with infectious secretions or excretions or through inhalation of aerosols (55). It is, therefore, possible that the transported beef steers already positive to this pathogen at T0, which were free to move inside the vehicles, may have expressed more exploratory behaviors and social interactions when transported at low stocking densities and during the stop, thus increasing the frequency of contacts between animals and the spreading of *P. multocida*. The presence of an extra stop during the journey should also be seen as an increase in journey duration and as an unuseful delay. It is well-known indeed that travel duration is a risk factor that increases the likelihood of opportunistic infections, such as Pasteurellaceae, in the lower respiratory tract (56) and of pneumonia in the transported animals (28, 57). Finally, MCA highlighted a cluster of animals where *P. multocida* positivity seemed to be related to BRSV positivity, spring season, and others as the category for the farm of arrival. In our study, the presence of BRSV was positively associated with *M. haemolytica*. This is in line with the literature because BRSV acts as a primary pathogen and predisposes the animal to secondary infections by *Pasteurella* spp. (14, 58) and *M. haemolytica* (59). As for *Pasteurella* spp., *M. haemolytica* positivity was more common at T1 among the animals that were subjected to an extra stop during the transport. However, this bacterium resulted to be positively associated also with low precipitations at arrival. A dry climate could be a possible predisposing factor because of the greater presence of dust in the air. This result seems to be consistent also with the evidence by Dubrovsky et al. (60) that reported dust occurring regularly in the calf-raising area was associated with increased BRD prevalence in pre-weaned calves. Dust may cause an inflammatory response in the nasopharyngeal tract, but also may be a carrier of airborne microorganisms, including viruses (61).

The exact role of viruses in BRD occurrence is still unclear. Viruses may produce a clinical syndrome consistent with BRD also in the absence of bacterial co-infection (62), but viral infections are generally considered as previous to, or concurrent with, bacterial infection (15). In our study, no positive DNS at departure and very few positivities at arrival were observed for BVDV, BoHV-1, and BPIV-3. On the other hand, BCoV was the pathogen showing the most dramatic increase from T0 and T1. This result suggests a fast replication of BCoV in the upper respiratory tract of animals in distress after long-distance transport, in agreement with the literature (14). Despite the significant increase, no associations were found. This result suggests that BCoVs spread independently of season, stocking densities, and climate, being extremely contagious no matter the travel and weather conditions. Contrariwise, BRSV resulted to be associated with an extra stop during transport, a particular farm (F3 and F4), a humidity ranging between 60 and 70%, and abnormally high temperatures in winter. This could be due to the fact that air humidity is considered among the main environmental risk factors increasing pathogen density and survival time outside the host (63). As observed for *P. multocida*, lower stocking densities during transport were associated with increased odds of having beef steers positive to BAdV. As already suggested, this may be related to the fact that increased space allowance during transport facilitates social interactions. However, during low stocking density, ventilation may also be stronger, increasing the spreading of the virus. The farm of arrival was associated with BAdV suggesting the epidemiological role of the farm of arrival even if our animals were isolated. Therefore, as the sampled animals did not get in touch with the others already located in the arrival farms, it may be likely that equipment and workers may have played roles. However, this hypothesis needs to be confirmed by further studies.

In our study, the odds of having beef steers with lacrimal and nasal discharges were higher at F2. Nasal discharge was significantly associated with the prevalence of *M. haemolytica* and BCoV, and with AT above 10°C. Both the presence of lacrimal discharge and diarrhea were associated with a higher AH. These results are therefore in agreement with the general view that sudden changes in air humidity represent a stressor possibly triggering a clinical state, and that high humidity favors dampness and disease spread. In addition, high ΔT and ΔH resulted in increase in the possibility of transported animals displaying diarrhea and coughing. These results are not surprising when we consider the scientific literature. Several viral agents may survive longer in aerosol with increased relative humidity in the air, and their persistence may also depend on temperature (64). Coughing was also more frequent if no arrival precipitations were noticed. As mentioned before, a dry climate may cause increased amounts of dust in the air, possibly causing an inflammatory response in the respiratory tract epithelium and also playing a role as a carrier of airborne microorganisms. Concerning coughing, this symptom was associated also with higher stocking density during transport. The more the animals, the more the concentration of noxious gases, impurities, and water vapor increases in the air during transport. This may participate in causing inflammatory reactions in the mucosa of the respiratory tract. However, coughing did not result to be associated with any positivity for the tested pathogens. This was expected since this clinical sign is non-specific, and it is not always present in the case of transport pneumonia in both horses (5) and bovine (33). Our result suggests that using cough monitoring to identify an early stage of BRD may not be appropriate. In the present study, the nasal discharge was instead found to be often associated with specific pathogens positivities. Thus, the presence of nasal discharge may be a more accurate marker to start treating the animals.

Antimicrobial metaphylaxis upon feedlot arrival is commonly used to reduce morbidity and mortality (65). However, this mass administration of antibiotics dramatically contributes to the development of antibiotic resistance, one of the major global health threats (66). To limit the spreading of pathogens linked to the transport of live animals and slow the development of antibiotic resistance, it would be desirable to identify more accurate diagnostic methods to test the animals before departure and other types of preventions. For instance, in our study before departure, many animals resulted to be positive but asymptomatic. The use of accurate diagnostic tools may help in identifying the animals positive to the BRD-related pathogens, preventing them from being transported, and decreasing the risks of pathogen spreading during and after transport. Given the high prevalence of BCoV at T1, it is also advisable to vaccinate animals before departure, to avoid the possibility that this pathogen may act as a trigger to other secondary bacterial infections involved in BRD occurrence. Based on our findings, immunization toward the pathogens present in the destination farms seems also a good practice. Good management practices, such as avoiding abrupt changes in the environmental parameters and the presence of dust inside the feedlot, may help to prevent the outbreak of BRD and should be also be recommended. Better transport practices would also help to reduce the use of antibiotics, thus limiting the exposure of bacteria to antibiotics, therefore slowing the development of antibiotic resistance (14).

Our results need to be interpreted with caution because the study has some limitations. First, this is a preliminary and an opportunistic research, and thus we had not the possibility to balance throughout the year of sampling all the variables possibly affecting BRD-related symptoms and pathogens. For example, as can be noticed by the MCA biplots, some farms received few animals and mainly in one or two seasons. This may have affected the power of the statistical analysis and might have been a confounding factor. Second, it was not possible to download from the vehicles and analyze the data concerning the temperatures and humidity conditions during transport, which may have contributed to explaining part of the variability observed in the considered sample. Third, mortality and morbidity rates of the total population (*n* = 1,045) were not recorded, and no other follow-up of the 169 tested beef steers was possible due to logistic problems. Fourth, since only nasal swabs were collected in unhandled animals and some sampling was difficult to perform, it would have been possible that our prevalence was underestimated due to false negative. Fifth, it was impossible to record the real duration of each journey and the rectal temperature. Finally, our methodological approach did not presume to define direct cause–effect relationships, but mainly to investigate possible associations useful to screen possible factors that need further investigation. Notwithstanding these limitations, this manuscript documents for the first time the prevalence of clinical signs and pathogens related to BRD in a consistent sample of beef steers (169 animals) throughout the whole year of long-distance travel from France to Italy with integrated use of regression and multivariate analyses.

Overall, the tested journey led to an increased prevalence of BRD-related pathogens and clinical signs, and we suggested that weather and travel conditions play important roles. It seems that environmental parameters and their abrupt changes, such as those occurring during hot seasons in Italy, are more likely to increase the prevalence of several symptoms and pathogens related to BRD. This paper also raises doubts about the effects of more than one unloading stop during long journeys that seemed to increase the positivity to particular bacteria. Finally, our results confirmed the importance of feedlot management as a factor affecting the prevalence of several BRD-related pathogens, suggesting that more interventions (i.e., more testing and immunizations) should be performed before transport.

## Data Availability Statement

The raw data supporting the conclusions of this article will be made available by the authors, without undue reservation.

## Ethics Statement

The experimental procedures were approved by the Ethics Committee of the Department of Veterinary Medicine of the University of Bari, Italy (authorization no. 16/18).

## Author Contributions

BP, GF, AG, AP, and DT: conceptualization. FC, BP, GF, AG, LAN, AP, and DT: methodology. BP and MZ: formal analysis. AP, FC, and MZ: investigation. AP: resources, supervision, and project administration. BP and MZ: data curation and writing—original draft preparation. BP and AP: writing—review and editing. All authors contributed to the article and approved the submitted version.

## Conflict of Interest

GF was employed by the company Siciliani S.p.A. Industria Lavorazione Carne. The remaining authors declare that the research was conducted in the absence of any commercial or financial relationships that could be construed as a potential conflict of interest.
