# Supplementary material for: Factors Affecting the Development of Bovine Respiratory Disease: A Cross-Sectional Study in Beef Steers Shipped From France to Italy
Source: Front Vet Sci. 2021 Jun 28;8:627894. doi: 10.3389/fvets.2021.627894 (PMC8273259; doi:10.3389/fvets.2021.627894)
Supplement: Supplementary file 1 [file Table_1.DOCX]

Supplementary Material

# Supplementary Figures and Tables

**Supplementary Table 1**. Primer and Real-Time quantitative PCR (RT-qPCR) probe sets for the detection of BRD-related pathogens in the DNS collected from 169 beef steers transported from France to Italy in one-year cross-sectional study.

| **Pathogens** | **Forward Primer** | **Reverse Primer** | **FAM** |
| --- | --- | --- | --- |
| **BPIV-3** | 5’-TGTCTTCCACTAGATAGA-3’ | 5’-GCAATGATAACAGGACTA-3’ | 5’-ACAGCAATTGGATCAATA[MGBEQ] -3’ |
| **BVDV** | 5’-GGGNAGTCGTCARTGGT-3’ | 5’-GTGCCCATGTACAGCAGAGWTTTT-3’ | 5’-CCAYGTGGACGAGGGCAYGC[TAM] -3’ |
| **BCoV** | 5’-GGACCCAAGTAGCGATGAG-3’ | 5’-GACCTTCCTGAGCCTTCAATA-3’ | 5’-ATTCCGACTAGGTTTCCGCCTGG[TAM] -3’ |
| **BRSV** | 5’-GCAATGCTGCAGGACTAGGTATAAT | 5’-ACACTGTAATTGATGACCCCATTCT-3’ | 5’-ACCAAGACTTGTATGATGCTGCCAAAGCA[TAM] -3’ |
| **BoHV-1** | 5’-CAATAACAGCGTAGACCTGGTC-3’ | 5’-GCTGTAGTCCCAAGCTTCCAC-3’ | 5’-TGCGGCCTCCGGGCTTTACGTCT[TAM] -3’ |
| **BAdV** | 5’-ATTACCAGCGTCAACCTCTAC-3’ | 5’-CCGCCGAGAGATAGTCATTAAA-3’ | 5’-TCCACTTTGGAAGCTATGCTCCGC[TAM] -3’ |
| ***M. haemolytica*** | 5’-ATTAGTGGGTTGTCCTGGTTAG-3’ | 5’-GCGTGATTTCGGTTCAGTTG-3’ | 5’-CTGAACCAACACGAGTAGTCGCTGC[TAM] -3’ |
| ***P. multocida*** | 5’-GGGCTTGTCGGTAGTCTTT-3’ | 5’-CGGCAAATAACAATAAGCTGAGTA | 5’-CGGCGCAACTGATTGGACGTTATT[TAM] -3’ |
| ***H. somni*** | 5’-AAGGCCTTCGGGTTGTAAAG-3’ | 5’-CCGGTGCTTCTTCTGTGATTAT-3’ | 5’-CGGTGATGAGGAAGGCGATTAG[TAM] -3’ |
| ***M. bovis*** | 5’-TCAAGGAACCCCACCAGAT-3’ | 5’-AGGCAAAGTCATTTCTAGGTGCAA-3’ | 5’-TGGCAAACTTACCTATCGGTGACCCT[TAM] -3’ |

**Supplementary Table 2**. The weather conditions recorded from a weather website in La Souterraine and in Naves (France) on the day of departure, and in Bari (Italy) on the day of arrival of the 169 beef steers considered in the one-year cross-sectional study.

|  | **Departure** | | | |  | **Arrival** | | | |
| --- | --- | --- | --- | --- | --- | --- | --- | --- | --- |
|  | **Autumn** | **Winter** | **Spring** | **Summer** |  | **Autumn** | **Winter** | **Spring** | **Summer** |
| Minimum Temperature (°C) | 2 | -1 | 1 | 12 |  | 7 | 3 | 6 | 18 |
| Temperature (°C) | 9 | 9 | 9 | 22 |  | 15 | 10 | 14 | 24 |
| Maximum Temperature (°C) | 18 | 16 | 16 | 35 |  | 25 | 18 | 24 | 29 |
| Minimum Humidity (%) | 60 | 60 | 50 | 45 |  | 65 | 60 | 65 | 60 |
| Humidity (%) | 82 | 78 | 60 | 59 |  | 73 | 76 | 78 | 73 |
| Maximum Humidity (%) | 100 | 85 | 75 | 80 |  | 85 | 80 | 85 | 80 |
| Minimum Wind Speed (km/h) | 8 | 8 | 12 | 6 |  | 10 | 10 | 13 | 10 |
| Wind Speed (km/h) | 19 | 19 | 18 | 14 |  | 15 | 18 | 15 | 14 |
| Maximum Wind Speed (km/h) | 40 | 41 | 40 | 25 |  | 30 | 57 | 26 | 25 |
| Minimum atmospheric precipitations (mm) | 0 | 0 | 0 | 0 |  | 0 | 0 | 0 | 0 |
| Atmospheric precipitations (mm) | 6 | 4 | 4 | 0 |  | 1 | 1 | 1 | 0 |
| Maximum atmospheric precipitations (mm) | 14 | 12 | 16 | 1 |  | 2 | 8 | 2 | 0 |

**Supplementary Table 3.** Frequency table of the animals (169 beef steers) transported from France to Italy for the variables related to weather conditions.

| **Variable** | **Count** | **Percent** |
| --- | --- | --- |
| Season |  |  |
| Autumn | 40 | 23.7 |
| Winter | 52 | 30.8 |
| Spring | 20 | 11.8 |
| Summer | 57 | 33.7 |
| Total | 169 | 100.0 |
| Arrival temperature (AT) |  |  |
| 1 (from 5 to 9°C) | 22 | 13.0 |
| 2 (from 10 to 13°C) | 50 | 29.6 |
| 3 (from 14 to 19°C) | 30 | 17.8 |
| 4 (from 20 to 23°C) | 23 | 13.6 |
| 5 (from 24 to 30°C) | 44 | 26.0 |
| Total | 169 | 100.0 |
| Diurnal temperature variation |  |  |
| 1 (from 0 to 5°C) | 36 | 21.3 |
| 2 (from 6 to 8°C) | 69 | 40.8 |
| 3 (from 9 to 11°C) | 49 | 29.0 |
| 4 (from 12 to 17°C) | 15 | 8.9 |
| Total | 169 | 100.0 |
| Delta temperature between arrival and departure (ΔT) |  |  |
| Low difference (from -1 to +3°C) | 84 | 49.7 |
| Medium difference (from +4 to +6°C) | 65 | 38.5 |
| High difference (from +7 to +10°C) | 20 | 11.8 |
| Total | 169 | 100.0 |
| Delta humidity between arrival and departure (ΔH) |  |  |
| 1 (from -35 to -10%) | 50 | 29.6 |
| 2 (from -5 to +10%) | 62 | 36.7 |
| 3 (from +15 to +30%) | 57 | 33.7 |
| Total | 169 | 100.0 |
| Arrival humidity (AH) |  |  |
| Medium-low (from 60 to 70%) | 63 | 37.3 |
| Medium-high (from 71 to 80%) | 91 | 53.8 |
| Very high (from 81 to 100%) | 15 | 8.9 |
| Total | 169 | 100.0 |
| Arrival wind speed |  |  |
| Low (from 0 to 10 km/h) | 45 | 26.6 |
| Medium-low (from 11 to 14 km/h) | 42 | 24.8 |
| Medium (from 15 to 29 km/h) | 36 | 21.3 |
| Strong (from 20 to 24 km/h) | 28 | 16.6 |
| Very strong (≥25 km/h) | 18 | 10.7 |
| Total | 169 | 100.0 |
| Arrival precipitations |  |  |
| No (0 mm) | 132 | 78.1 |
| Yes (from 0.5 to 8 mm) | 37 | 21.9 |
| Total | 169 | 100.0 |

**Supplementary Table 4.** Associations between the predictive variables (weather and transport conditions) and the presence/absence of clinical signs. The symptoms were observed four days after arrival (T1) in 169 beef steers transported from France to Italy. Associations are presented with Wald Test *p*-values.

| **Predictive variables** | **Wald Test *p*-value** |
| --- | --- |
| **Coughing at T1** | |
| Season | 0.005 |
| Arrival temperature (AT) | 0.009 |
| Arrival humidity (AH) | 0.848 |
| Diurnal temperature variation | <0.001 |
| Delta temperature between arrival and departure (ΔT) | 0.053 |
| Delta humidity between arrival and departure (ΔH) | 0.079 |
| Arrival wind speed | 0.150 |
| Arrival precipitations | 0.086 |
| Stocking density | 0.031 |
| Extra stop | 0.985 |
| Farm (F) | 0.311 |
| **Diarrhea at T1** | |
| Season | 0.784 |
| Arrival temperature (AT) | 0.443 |
| Arrival humidity (AH) | 0.024 |
| Diurnal temperature variation | <0.001 |
| Delta temperature between arrival and departure (ΔT) | <0.001 |
| Delta humidity between arrival and departure (ΔH) | 0.029 |
| Arrival wind speed | 0.731 |
| Arrival precipitations | 0.644 |
| Stocking density | 0.619 |
| Extra stop | 0.331 |
| Farm (F) | 0.425 |
| **Lacrimal discharge at T1** | |
| Season | 0.040 |
| Arrival temperature (AT) | <0.001 |
| Arrival humidity (AH) | 0.011 |
| Diurnal temperature variation | 0.063 |
| Delta temperature between arrival and departure (ΔT) | 0.002 |
| Delta humidity between arrival and departure (ΔH) | 0.010 |
| Arrival wind speed | 0.950 |
| Arrival precipitations | 0.987 |
| Stocking density | 0.118 |
| Extra stop | 0.883 |
| Farm (F) | <0.001 |
| **Nasal discharge at T1** | |
| Season | 0.007 |
| Arrival temperature (AT) | <0.001 |
| Arrival humidity (AH) | 0.137 |
| Diurnal temperature variation | 0.964 |
| Delta temperature between arrival and departure (ΔT) | 0.235 |
| Delta humidity between arrival and departure (ΔH) | 0.050 |
| Arrival wind speed | 0.069 |
| Arrival precipitations | <0.001 |
| Stocking density | 0.854 |
| Extra stop | 0.024 |
| Farm (F) | 0.139 |

**Supplementary Table 5.** Significant associations between the predictive variables (weather and transport conditions) and the presence/absence of clinical signs. The symptoms were observed four days after arrival in 169 beef steers transported from France to Italy. Data are presented as odds ratio (OR), confidence interval (95% CI) and *p*-value (*p*).

| **Variable** | | **Category** | | **Univariable model** | | | | |
| --- | --- | --- | --- | --- | --- | --- | --- | --- |
|  |  |  |  | **OR** | | **95%CI** | ***p*** | |
| **Dependent variable: coughing** | | | | | | | | |
| Season | | Autumn | | ref | |  |  | |
|  |  | Winter | | 7.00 | | 1.79-46.54 | 0.014 | |
|  |  | Spring | | 2.11 | | 0.24-18.77 | 0.472 | |
|  |  | Summer | | 0.69 | | 0.08-5.96 | 0.717 | |
| Stocking density | | Low | | ref | |  |  | |
|  |  | High | | 3.54 | | 1.23-12.82 | 0.030 | |
| AT | | 5 (24 - 30°C) | | ref | |  |  | |
|  |  | 1 (5 - 9°C) | | 2.05 | | 0.08-53.51 | 0.618 | |
|  |  | 2 (10 - 13°C) | | 16.72 | | 3.13-310.34 | 0.008 | |
|  |  | 3 (14 - 19°C) | | 4.78 | | 0.58-99.31 | 0.185 | |
|  |  | 4 (20 - 23°C) | | 1.95 | | 0.07-51.02 | 0.641 | |
| Diurnal temperature variation | | 2 (6 - 8°C) | | ref | |  |  | |
|  |  | 1 (0 - 5°C) | | n.e. | | n.e. | 0.992 | |
|  |  | 3 (9 - 11°C) | | 1.44 | | 0.33-6.40 | 0.616 | |
|  |  | 4 (12 - 17°C) | | 65.00 | | 14.67-396.28 | < .001 | |
| **Dependent variable: diarrhea** | | | | | | | | |
| Diurnal temperature variation | 3 (9 - 11°C) | | ref | |  | |  |  |
|  | 1 (0 - 5°C) | | 15.33 | | 4.53-71.35 | | <0.001 |  |
|  | 2 (6 - 8°C) | | 1.20 | | 0.28-6.07 | | 0.811 |  |
|  | 4 (12 - 17°C) | | 1.09 | | 0.05-9.35 | | 0.939 |  |
| ΔT | Low | | ref | |  | |  |  |
|  | Medium | | 1.53 | | 0.55-4.30 | | 0.413 |  |
|  | High | | 9.50 | | 3.09-30.92 | | <0.001 |  |
| AH | Medium-low | | ref | |  | |  |  |
|  | Medium-high | | 3.57 | | 1.25-12.89 | | 0.026 |  |
|  | Very high | | 7.25 | | 1.66-34.10 | | 0.008 |  |
| ΔH | Medium | | ref | |  | |  |  |
|  | Low | | 5.55 | | 1.61-25.69 | | 0.012 |  |
|  | High | | 5.81 | | 1.75-26.47 | | 0.009 |  |

| **Dependent variable: lacrimal discharge** | | | | | | | | | |
| --- | --- | --- | --- | --- | --- | --- | --- | --- | --- |
| F | F1 | | ref | |  | |  | |  |
|  | F2 | | 21.18 | | 6.02-134.61 | | <0.001 | |  |
|  | Others | | n.e. | | n.e. | | 0.989 | |  |
| Season | Spring | | ref | |  | |  | |  |
|  | Autumn | | n.e. | | n.e. | | 0.995 | |  |
|  | Winter | | n.e. | | n.e. | | 0.994 | |  |
|  | Summer | | 10.00 | | 2.57-66.64 | | 0.004 | |  |
| AT | 2 (10 - 13°C) | | ref | |  | |  | |  |
|  | 1 (5 - 9°C) | | n.e. | | n.e. | | 0.991 | |  |
|  | 3 (14 - 19°C) | | 1.69 | | 0.06-43.82 | | 0.714 | |  |
|  | 4 (20 - 23°C) | | 2.23 | | 0.08-5.81 | | 0.577 | |  |
|  | 5 (24 - 30°C) | | 94.73 | | 17.94-1759.37 | | <0.001 | |  |
| ΔT | Medium difference | | ref | |  | |  | |  |
|  | Low difference | | 7.62 | | 2.78-26.96 | | <0.001 | |  |
|  | High difference | | n.e. | | n.e. | | 0.991 | |  |
| AH | Medium-low | | ref | |  | |  | |  |
|  | Medium-high | | 4.89 | | 1.90-15.18 | | 0.002 | |  |
|  | Very high | | n.e. | | n.e. | | 0.988 | |  |
| ΔH | 1 (-35 to -10%) | | ref | |  | |  | |  |
|  | 2 (-5 to +10%) | | 13.00 | | 2.45- 240.70 | | 0.015 | |  |
|  | 3 (+15 to +30%) | | 22.61 | | 4.37- 415.79 | | 0.003 | |  |
| **Dependent variable: nasal discharge** | | | | | | | | | |
| Season | Autumn | ref | |  | |  | |  | |
|  | Winter | 2.72 | | 1.16-6.65 | | 0.024 | |  | |
|  | Spring | 3.50 | | 1.16-11.15 | | 0.029 | |  | |
|  | Summer | 4.67 | | 1.99-11.50 | | <0.001 | |  | |
| Extra stop | No | ref | |  | |  | |  | |
|  | Yes | 2.21 | | 1.13-4.47 | | 0.023 | |  | |
| AT | 1 (5 - 9°C) | ref | |  | |  | |  | |
|  | 2 (10 - 13°C) | 2.27 | | 0.76-7.79 | | 0.162 | |  | |
|  | 3 (14 - 19°C) | 9.35 | | 2.75-36.97 | | <0.001 | |  | |
|  | 4 (20 - 23°C) | 3.12 | | 0.89-12.15 | | 0.084 | |  | |
|  | 5 (24 - 30°C) | 9.07 | | 2.90-32.88 | | <0.001 | |  | |
| Arrival precipitations | Yes | ref | |  | |  | |  | |
|  | No | 6.06 | | 2.68-15.20 | | <0.001 | |  | |

n.e. stands for not estimable.

**Supplementary Table 6.** Associations between the predictive variables (weather and transport conditions, positivity for a virus at departure-T0) and the presence/absence of viral infections. The positivities were observed four days after arrival in 169 beef steers transported from France to Italy. Associations are presented with Wald Test *p*-values.

| **Predictive variables** | **Wald Test *p*-value** |
| --- | --- |
| **BRSV at T1** | |
| Season | 0.101 |
| Arrival temperature (AT) | 0.345 |
| Arrival humidity (AH) | 0.009 |
| Diurnal temperature variation | 0.108 |
| Delta temperature between arrival and departure (ΔT) | 0.395 |
| Delta humidity between arrival and departure (ΔH) | 0.899 |
| Arrival wind speed | 0.005 |
| Arrival precipitations | 0.409 |
| Stocking density | 0.004 |
| Extra stop | <0.001 |
| Farm (F) | 0.003 |
| BRSV positivity at T0 | 0.990 |
| **BAdV at T1** | |
| Season | 0.150 |
| Arrival temperature (AT) | 0.286 |
| Arrival humidity (AH) | 0.001 |
| Diurnal temperature variation | 0.702 |
| Delta temperature between arrival and departure (ΔT) | 0.283 |
| Delta humidity between arrival and departure (ΔH) | 0.005 |
| Arrival wind speed | 0.059 |
| Arrival precipitations | 0.222 |
| Stocking density | 0.005 |
| Extra stop | 0.398 |
| Farm (F) | 0.017 |
| BAdV positivity at T0 | 0.019 |
| **BCoV at T1** | |
| Season | 0.284 |
| Arrival temperature (AT) | 0.749 |
| Arrival humidity (AH) | 0.214 |
| Diurnal temperature variation | 0.910 |
| Delta temperature between arrival and departure (ΔT) | 0.847 |
| Delta humidity between arrival and departure (ΔH) | 0.995 |
| Arrival wind speed | 0.213 |
| Arrival precipitations | 0.233 |
| Stocking density | 0.786 |
| Extra stop | 0.096 |
| Farm (F) | 0.995 |
| BCoV positivity at T1 | 0.531 |

**Supplementary Table 7.** Significant associations between the predictive variables (weather and transport conditions, positivity for a virus at departure-T0) and the presence/absence of viral infections. The positivities were observed four days after arrival in 169 beef steers transported from France to Italy. Data are presented as odds ratio (OR), confidence interval (95% CI) and *p*-value (*p*).

| **Variable** | | | **Category** | **Univariate** | | | |
| --- | --- | --- | --- | --- | --- | --- | --- |
|  |  |  |  | **OR** | **95%CI** | ***p*** | |
| **Dependent variable: BRSV positivity** | | | | | | |  |
| F | | | F2 | ref |  |  | |
|  |  |  | F1 | 4.79 | 1.15-32.46 | 0.053 | |
|  |  |  | Others | 21.94 | 4.11-170.11 | <0.001 | |
| Arrival wind speed | | | Medium-low | ref |  |  | |
|  |  |  | Low | n.e. | n.e. | 0.992 | |
|  |  |  | Medium | 6.61 | 1.00-130.14 | 0.092 | |
|  |  |  | Strong | 1.52 | 0.06-39.50 | 0.771 | |
|  |  |  | Very strong | 32.80 | 5.20-646.26 | 0.002 | |
| Stocking density | | | High | ref |  |  | |
|  |  |  | Low | 9.91 | 2.62-64.81 | 0.003 | |
| Extra stop | | | No | ref |  |  | |
|  |  |  | Yes | 11.79 | 3.53-53.76 | <0.001 | |
| AH | | | Medium-high | ref |  |  | |
|  |  |  | Medium-low | 25.71 | 4.95-472.93 | 0.002 | |
|  |  |  | Very high | n.e. | n.e. | 0.993 | |
| **Dependent variable: BAdV positivity** | | | | | | |  |
| F | F1 | | | ref |  |  | |
|  | F2 | | | 13.96 | 2.67-256.92 | 0.012 | |
|  | Others | | | 29.20 | 3.86-603.10 | 0.004 | |
| Stocking density | High | | | ref |  |  | |
|  | Low | | | 0.19 | 0.05-0.55 | 0.005 | |
| ΔH | | 1 (-35 to -10%) | | ref |  |  | |
|  |  | 2 (-5 to +10%) | | 3.38 | 0.48-67.28 | 0.283 | |
|  |  | 3 (+15 to +30%) | | 14.48 | 2.71-268.34 | 0.012 | |
| BAdV positivity at T0 | No | | | ref |  |  | |
|  | Yes | | | 18.75 | 1.71-416.94 | 0.019 | |
| AH | Medium-high | | | ref |  |  | |
|  | Medium-low | | | 4.35 | 1.20-20.49 | 0.021 | |
|  | Very high | | | 19.55 | 4.42-106.36 | <0.001 | |

**Supplementary Table 8.** Associations between the predictive variables (weather and transport conditions, positivity for a bacterial infection at departure-T0, positivity for a virus at T1) and the presence/absence of bacterial infections. The positivities were observed four days after arrival in 169 beef steers transported from France to Italy. Associations are presented with Wald Test *p*-values.

| **Predictive variables** | **Wald Test *p*-value** |
| --- | --- |
| ***H. somni* positivity at T1** | |
| Season | <0.001 |
| Arrival temperature (AT) | 0.078 |
| Arrival humidity (AH) | 0.438 |
| Diurnal temperature variation | <0.001 |
| Delta temperature between arrival and departure (ΔT) | 0.686 |
| Delta humidity between arrival and departure (ΔH) | 0.010 |
| Arrival wind speed | 0.239 |
| Arrival precipitations | 0.737 |
| Stocking density | 0.988 |
| Extra stop | 0.990 |
| Farm (F) | 0.008 |
| *H. somni* positivity at T0 | 0.137 |
| BRSV positivity at T1 | 0.489 |
| BAdV positivity at T1 | 0.991 |
| BCoV positivity at T1 | 0.744 |
| ***M. haemolytica* positivity at T1** | |
| Season | 0.457 |
| Arrival temperature (AT) | 0.017 |
| Arrival humidity (AH) | 0.606 |
| Diurnal temperature variation | 0.098 |
| Delta temperature between arrival and departure (ΔT) | 0.047 |
| Delta humidity between arrival and departure (ΔH) | 0.481 |
| Arrival wind speed | 0.121 |
| Arrival precipitations | 0.037 |
| Stocking density | 0.033 |
| Extra stop | 0.084 |
| Farm (F) | 0.291 |
| *M. haemolytica* positivity at T0 | 0.189 |
| BRSV positivity at T1 | 0.004 |
| BAdV positivity at T1 | 0.159 |
| BCoV positivity at T1 | 0.060 |
| ***M. bovis* positivity at T1** | |
| Season | <0.001 |
| Arrival temperature (AT) | 0.023 |
| Arrival humidity (AH) | 0.522 |
| Diurnal temperature variation | 0.094 |
| Delta temperature between arrival and departure (ΔT) | 0.007 |
| Delta humidity between arrival and departure (ΔH) | <0.001 |
| Arrival wind speed | <0.001 |
| Arrival precipitations | 0.147 |
| Stocking density | 0.111 |
| Extra stop | 0.237 |
| Farm (F) | 0.009 |
| *M. bovis* positivity at T0 | 0.191 |
| BRSV positivity at T1 | 0.013 |
| BAdV positivity at T1 | 0.515 |
| BCoV positivity at T1 | 0.126 |
| ***P. multocida* positivity at T1** | |
| Season | 0.005 |
| Arrival temperature (AT) | 0.069 |
| Arrival humidity (AH) | 0.568 |
| Diurnal temperature variation | 0.538 |
| Delta temperature between arrival and departure (ΔT) | 0.900 |
| Delta humidity between arrival and departure (ΔH) | 0.830 |
| Arrival wind speed | 0.021 |
| Arrival precipitations | 0.409 |
| Stocking density | <0.001 |
| Extra stop | <0.001 |
| Farm (F) | 0.338 |
| *P. multocida* positivity at T0 | <0.001 |
| BRSV positivity at T1 | 0.140 |
| BAdV positivity at T1 | 0.187 |
| BCoV positivity at T1 | 0.068 |

**Supplementary Table 9.** Significant associations between the predictive variables (weather and transport conditions, positivity for a bacterial infection at departure-T0, positivity for a virus at T1) and the presence/absence of bacterial infections. The positivities were observed four days after arrival in 169 beef steers transported from France to Italy. Data are presented as odds ratio (OR), confidence interval (95% CI) and *p*-value (*p*).

| **Variable** | | | | | | **Category** | **Univariate** | | |
| --- | --- | --- | --- | --- | --- | --- | --- | --- | --- |
|  |  |  |  |  |  |  | **OR** | **95%CI** | ***p*** |
| **Dependent variable: *H. somni* positivity at T1** | | | | | | | | | |
| Season | Winter | | | | | | ref |  |  |
|  | Autumn | | | | | | 20.65 | 3.94-381.19 | 0.004 |
|  | Spring | | | | | | n.e. | n.e. | 0.990 |
|  | Summer | | | | | | 14.56 | 3.88-95.27 | <0.001 |
| F | F2 | | | | | | ref |  |  |
|  | F1 | | | | | | 11.03 | 3.04-70.99 | 0.002 |
|  | Others | | | | | | n.e. | n.e. | 0.992 |
| ΔH | | 2 (-5 to +10%) | | | | | ref |  |  |
|  |  | 1 (-35 to -10%) | | | | | n.e. | n.e. | 0.990 |
|  |  | 3 (+15 to+30%) | | | | | 7.53 | 2.36-33.68 | 0.002 |
| Diurnal temperature variation | | 4 (12 - 17°C) | | | | | ref |  |  |
|  |  | 1 (0 - 5°C) | | | | | 22.00 | 4.96-128.83 | <0.001 |
|  |  | 2 (6 - 8°C) | | | | | 25.60 | 6.71-116.23 | <0.001 |
|  |  | 3 (9 - 11°C) | | | | | 30.67 | 7.01-178.14 | <0.001 |
| **Dependent variable: *M. haemolytica* positivity at T1** | | | | | | | | | |
| Stocking density | | | High | | | | ref |  |  |
|  |  |  | Low | | | | 2.53 | 1.10-6.12 | 0.032 |
| Diurnal temperature variation | | | | | 3 (9 - 11°C) | | ref |  |  |
|  |  |  |  |  | 1 (0 - 5°C) | | 1.96 | 0.71-5.51 | 0.194 |
|  |  |  |  |  | 2 (6 - 8°C) | | 0.50 | 0.17-1.45 | 0.204 |
|  |  |  |  |  | 4 (12 - 17°C) | | n.e. | n.e. | 0.987 |
| ΔT | | | | Medium | | | ref |  |  |
|  |  |  |  | Low | | | 2.82 | 1.04-9.05 | 0.055 |
|  |  |  |  | High | | | 5.14 | 1.37-20.29 | 0.015 |
| AT | | | 2 (10 - 13°C) | | | | ref |  |  |
|  |  |  | 3 (14 - 19°C) | | | | 3.92 | 0.95-19.86 | 0.068 |
|  |  |  | 4 (20 - 23°C) | | | | 12.05 | 3.17-59.96 | <0.001 |
|  |  |  | 5 (24 - 30°C) | | | | 3.48 | 0.93-16.76 | 0.079 |
|  |  |  | 1 (5 - 9°C) | | | | n.e. | n.e. | 0.990 |
| Arrival precipitations | | | Yes | | | | ref |  |  |
|  |  |  | No | | | | 8.91 | 1.79-161.95 | 0.035 |
| BRSV positivity at T1 | | | No | | | | ref |  |  |
|  |  |  | Yes | | | | 5.86 | 1.87-18.14 | 0.002 |

| **Dependent variable: *M. bovis* positivity at T1** | | | | |  |
| --- | --- | --- | --- | --- | --- |
| Season | Winter | ref |  |  |  |
|  | Autumn | 16.46 | 6.14-49.10 | <0.001 |  |
|  | Spring | 4.78 | 1.56-15.33 | 0.007 |  |
|  | Summer | 4.61 | 1.96-11.70 | <0.001 |  |
| F | F2 | ref |  |  |  |
|  | F1 | 2.81 | 1.47-5.44 | 0.002 |  |
|  | Others | n.e. | n.e. | 0.987 |  |
| AT | 1 (5 - 9°C) | ref |  |  |  |
|  | 2 (10 - 13°C) | 6.21 | 1.98-23.97 | 0.003 |  |
|  | 3 (14 - 19°C) | 3.94 | 1.14-16.18 | 0.039 |  |
|  | 4 (20 - 23°C) | 7.00 | 1.91-30.80 | 0.005 |  |
|  | 5 (24 - 30°C) | 2.83 | 0.88-11.09 | 0.100 |  |
| ΔT | High difference | ref |  |  |  |
|  | Low difference | 4.46 | 1.37-20.14 | 0.024 |  |
|  | Medium difference | 7.97 | 2.39-36.62 | 0.002 |  |
| ΔH | 2 (-5 to +10%) | ref |  |  |  |
|  | 1 (-35 to -10%) | 3.37 | 1.54-7.62 | 0.003 |  |
|  | 3 (+15 to+30%) | 4.57 | 2.13-10.20 | <0.001 |  |
| Arrival wind speed | Medium-low | ref |  |  |  |
|  | Low | 2.28 | 0.95-5.70 | 0.070 |  |
|  | Medium | 3.12 | 1.24-8.19 | 0.017 |  |
|  | Strong | 20.83 | 5.96-99.83 | <0.001 |  |
|  | Very strong | n.e. | n.e. | 0.986 |  |
| BRSV positivity at T1 | Yes | ref |  |  | |
|  | No | 14.00 | 2.71-256.81 | 0.012 | |
| **Dependent variable: *P. multocida* positivity at T1** | | | | | |
| Season | Autumn | ref |  |  | |
|  | Winter | 1.33 | 0.53-3.45 | 0.543 | |
|  | Spring | 9.00 | 2.76-33.95 | <0.001 | |
|  | Summer | 1.50 | 0.62-3.81 | 0.379 | |
| Stocking density | High | ref |  |  | |
|  | Low | 3.50 | 1.83-6.86 | <0.001 | |
| Extra stop | No | ref |  |  | |
|  | Yes | 4.76 | 2.38-9.73 | <0.001 | |
| *P. multocida* positivity at T0 | No | ref |  |  | |
|  | Yes | 7.56 | 2.77-24.30 | <0.001 | |
| Arrival wind speed | Strong | ref |  |  | |
|  | Low | 3.00 | 0.94-11.60 | 0.079 | |
|  | Medium-low | 2.69 | 0.83-10.53 | 0.119 | |
|  | Medium | 7.50 | 2.33-29.60 | 0.001 | |
|  | Very strong | 4.80 | 1.23-21.63 | 0.029 | |

**Supplementary Table 10**. The contribution of the variable categories (in %) to the definition of the dimensions of the Multiple Correspondence Analysis (MCA) performed on the categorical variables of clinical signs presence, pathogen positivity, travel and weather conditions, recorded in 169 beef steers transported from France to Italy. The inertia percentages explained by the dimensions are reported between brackets.

| **Categories** | **Dim1 (11.6%)** | **Dim2 (10.2%)** | **Dim3 (9.5%)** |
| --- | --- | --- | --- |
| AT = 5 | **7.64** | 1.74 | 0.00 |
| Season = Summer | **6.87** | **2.70** | 0.22 |
| Lacrimal discharge = Present | **6.84** | 1.11 | 0.00 |
| F = F2 | **6.71** | 0.62 | 0.08 |
| ΔT = medium | **5.38** | 0.05 | 0.01 |
| Season = Autumn | **4.98** | 0.07 | **8.56** |
| F = F1 | **4.89** | 0.05 | 1.46 |
| AT = 3 | **4.68** | 0.85 | **2.78** |
| *H.somni* at T0 = Positive | **4.17** | 0.35 | 0.13 |
| ΔT = low | **3.78** | 0.12 | 0.56 |
| AH = medium-high | **3.58** | 0.75 | 0.05 |
| ΔH = 1 | **3.02** | 0.78 | 0.79 |
| Arrival wind speed = strong | **2.94** | 0.01 | **2.95** |
| AH = medium-low | **2.75** | 0.46 | 0.77 |
| AT = 2 | **2.46** | 1.30 | 0.01 |
| *M.bovis* at T0 = Positive | **2.45** | 1.33 | 0.16 |
| Diurnal temperature variation = 3 | **2.11** | 0.01 | **4.30** |
| Diurnal temperature variation = 2 | 1.96 | 0.19 | 0.32 |
| Arrival precipitations = yes | 1.93 | 0.75 | 1.99 |
| *H.somni* at T0 = Negative | 1.66 | 0.14 | 0.05 |
| Lacrimal discharge = Absent | 1.60 | 0.26 | 0.00 |
| AH = very high | 1.58 | 0.56 | 1.56 |
| Season = Spring | 1.38 | **2.27** | **2.37** |
| F = Others | 1.31 | 1.87 | **4.46** |
| BRSV at T1 = Positive | 1.24 | 0.01 | **7.05** |
| *M.haemolytica* at T0 = Positive | 1.24 | 0.32 | 0.54 |
| Arrival wind speed = medium-low | 0.96 | **2.08** | 0.17 |
| ΔH = 2 | 0.86 | **6.93** | 1.85 |
| Stocking density = Low | 0.82 | **3.58** | 1.54 |
| AT = 1 | 0.77 | **6.77** | 0.04 |
| BCoV at T0 = Positive | 0.72 | 0.22 | 0.32 |
| Nasal discharge = Absent | 0.71 | 0.13 | 1.60 |
| Stocking density = High | 0.64 | **2.79** | 1.20 |
| Nasal discharge = Present | 0.62 | 0.11 | 1.40 |
| Arrival wind speed = very strong | 0.60 | 0.62 | **3.62** |
| Arrival wind speed = low | 0.57 | 0.06 | **3.55** |
| Arrival precipitations = no | 0.54 | 0.21 | 0.56 |
| ΔH = 3 | 0.47 | **3.57** | 0.32 |
| *H.somni* at T1 = Negative | 0.42 | **8.35** | 0.19 |
| *P.multocida* at T0 = Positive | 0.23 | **2.30** | 0.03 |
| Diurnal temperature variation = 4 | 0.17 | **3.05** | 1.06 |
| BRSV at T0 = Positive | 0.16 | 0.06 | 0.97 |
| *M.bovis* at T1 = Positive | 0.14 | 0.22 | 1.86 |
| BCoV at T0 = Negative | 0.14 | 0.04 | 0.06 |
| Extra stop = Yes | 0.12 | **3.59** | **8.08** |
| BRSV at T1 = Negative | 0.12 | 0.00 | 0.69 |
| *M.bovis* at T1 = Negative | 0.12 | 0.19 | 1.59 |
| *M.haemolytica* at T0 = Negative | 0.11 | 0.03 | 0.05 |
| *M.bovis* at T0 = Negative | 0.11 | 0.06 | 0.01 |
| *M.haemolytica* at T1 = Positive | 0.09 | **2.25** | 0.35 |
| Diarrhea = Present | 0.09 | **2.29** | 0.98 |
| Coughing = Present | 0.08 | **2.85** | 1.33 |
| BAdV at T1 = Positive | 0.07 | **3.77** | 0.06 |
| *P.multocida* at T1 = Positive | 0.07 | 1.62 | 1.31 |
| *H.somni* at T1 = Positive | 0.06 | 1.19 | 0.03 |
| Extra stop = No | 0.05 | 1.55 | **3.49** |
| ΔT = high | 0.04 | 1.27 | 1.75 |
| *P.multocida* at T1 = Negative | 0.04 | 0.89 | 0.72 |
| *P.multocida* at T0 = Negative | 0.03 | 0.33 | 0.00 |
| Arrival wind speed = medium | 0.03 | **5.21** | **3.37** |
| *M.haemolytica* at T1 = Negative | 0.02 | 0.43 | 0.07 |
| Diarrhea = Absent | 0.02 | 0.44 | 0.19 |
| Coughing = Absent | 0.01 | 0.38 | 0.18 |
| AT = 4 | 0.01 | 1.81 | **3.58** |
| BAdV at T1 = Negative | 0.01 | 0.45 | 0.01 |
| Season = Winter | 0.00 | **8.34** | **4.42** |
| BCoV at T1 = Negative | 0.00 | 0.16 | 0.62 |
| BCoV at T1 = Positive | 0.00 | 0.08 | 0.33 |
| BRSV at T0 = Negative | 0.00 | 0.00 | 0.01 |
| Diurnal temperature variation = 1 | 0.00 | 0.41 | **5.28** |
| BAdV at T0 = Positive | 0.00 | 0.64 | 0.02 |
| BAdV at T0 = Negative | 0.00 | 0.01 | 0.00 |

**Supplementary Table 11**. The coordinates of the variable categories in the dimensions of the MCA performed on the categorical variables of clinical signs presence, pathogen positivity, travel and weather conditions, recorded in 169 beef steers transported from France to Italy.

| **Categories** | **Dim1** | **Dim2** | **Dim3** |
| --- | --- | --- | --- |
| BRSV at T0 = Positive | **1.16** | 0.69 | **2.59** |
| AT = 3 | **1.15** | 0.46 | 0.80 |
| Season = Autumn | **1.03** | 0.11 | **-1.22** |
| AH = very high | **0.94** | 0.53 | -0.85 |
| Arrival wind speed = strong | **0.94** | 0.06 | **-0.85** |
| F = Others | **0.89** | **0.99** | **1.48** |
| BRSV at T1 = Positive | **0.84** | -0.06 | **1.80** |
| ΔT = medium | 0.84 | -0.08 | -0.04 |
| Season = Spring | 0.76 | **0.91** | **0.90** |
| F = F1 | 0.75 | 0.07 | -0.37 |
| ΔH = 1 | 0.71 | 0.34 | -0.33 |
| Arrival precipitations = yes | 0.66 | -0.39 | -0.61 |
| AT = 2 | 0.64 | -0.44 | -0.04 |
| AH = medium-low | 0.61 | 0.23 | 0.29 |
| Diurnal temperature variation = 3 | 0.60 | -0.04 | 0.78 |
| Arrival wind speed = very strong | 0.53 | -0.50 | **1.18** |
| *H.somni* at T0 = Negative | 0.34 | -0.09 | -0.05 |
| Lacrimal discharge = Absent | 0.31 | -0.12 | 0.00 |
| Diurnal temperature variation = 4 | 0.31 | **-1.23** | 0.70 |
| Stocking density = Low | 0.31 | 0.60 | 0.38 |
| Nasal discharge = Absent | 0.28 | -0.11 | -0.37 |
| Coughing = Present | 0.19 | **-1.03** | 0.68 |
| Extra stop = Yes | 0.14 | 0.72 | **1.05** |
| *M.bovis* at T1 = Positive | 0.12 | 0.15 | -0.41 |
| *P.multocida* at T1 = Positive | 0.10 | 0.45 | 0.39 |
| BCoV at T0 = Negative | 0.09 | -0.05 | -0.05 |
| *M.haemolytica* at T0 = Negative | 0.08 | -0.04 | 0.05 |
| *M.bovis* at T0 = Negative | 0.07 | -0.05 | -0.02 |
| *H.somni* at T1 = Positive | 0.06 | 0.24 | -0.04 |
| *P.multocida* at T0 = Negative | 0.04 | -0.13 | -0.01 |
| *M.haemolytica* at T1 = Negative | 0.03 | -0.15 | -0.06 |
| Diarrhea = Absent | 0.03 | -0.15 | 0.10 |
| Season = Winter | 0.02 | **-1.09** | 0.77 |
| BAdV at T1 = Negative | 0.02 | -0.15 | -0.02 |
| BCoV at T1 = Negative | 0.02 | -0.14 | -0.27 |
| BAdV at T0 = Negative | 0.00 | -0.02 | 0.00 |
| BRSV at T0 = Negative | -0.01 | 0.00 | -0.02 |
| BCoV at T1 = Positive | -0.01 | 0.07 | 0.15 |
| Diurnal temperature variation = 1 | -0.01 | 0.29 | **-1.01** |
| Coughing = Absent | -0.02 | 0.14 | -0.09 |
| BAdV at T0 = Positive | -0.05 | **1.25** | 0.22 |
| *P.multocida* at T1 = Negative | -0.05 | -0.25 | -0.21 |
| Extra stop = No | -0.06 | -0.31 | -0.45 |
| AT = 4 | -0.06 | 0.76 | **-1.04** |
| Arrival wind speed = medium | -0.08 | **1.03** | 0.80 |
| BRSV at T1 = Negative | -0.08 | 0.01 | -0.18 |
| *M.bovis* at T1 = Negative | -0.11 | -0.12 | 0.35 |
| ΔT = high | -0.13 | 0.68 | -0.78 |
| Diarrhea = Present | -0.17 | 0.79 | -0.50 |
| *M.haemolytica* at T1 = Positive | -0.17 | 0.78 | 0.30 |
| BAdV at T1 = Positive | -0.18 | **1.24** | 0.15 |
| Arrival precipitations = no | -0.19 | 0.11 | 0.17 |
| Stocking density = High | -0.24 | -0.47 | -0.30 |
| Nasal discharge = Present | -0.24 | 0.10 | 0.33 |
| ΔH = 3 | -0.26 | 0.68 | -0.20 |
| *P.multocida* at T0 = Positive | -0.30 | **0.90** | 0.10 |
| Arrival wind speed = low | -0.33 | -0.10 | -0.74 |
| ΔH = 2 | -0.35 | **-0.92** | 0.46 |
| *H.somni* at T1 = Negative | -0.41 | **-1.71** | 0.25 |
| Arrival wind speed = medium-low | -0.44 | -0.60 | 0.17 |
| BCoV at T0 = Positive | -0.48 | 0.24 | 0.29 |
| Diurnal temperature variation = 2 | -0.49 | 0.14 | -0.18 |
| AT = 1 | -0.54 | **-1.51** | 0.11 |
| AH = medium-high | -0.58 | -0.25 | -0.06 |
| ΔT = low | -0.62 | -0.10 | 0.21 |
| F = F2 | **-0.84** | -0.24 | 0.08 |
| *H.somni* at T0 = Positive | **-0.86** | 0.23 | 0.14 |
| *M.haemolytica* at T0 = Positive | **-0.87** | 0.41 | -0.52 |
| Season = Summer | **-1.01** | 0.59 | -0.16 |
| AT = 5 | **-1.21** | 0.54 | -0.02 |
| Lacrimal discharge = Present | **-1.34** | 0.51 | -0.01 |
| *M.bovis* at T0 = Positive | **-1.72** | **1.19** | 0.40 |

## Supplementary Figures


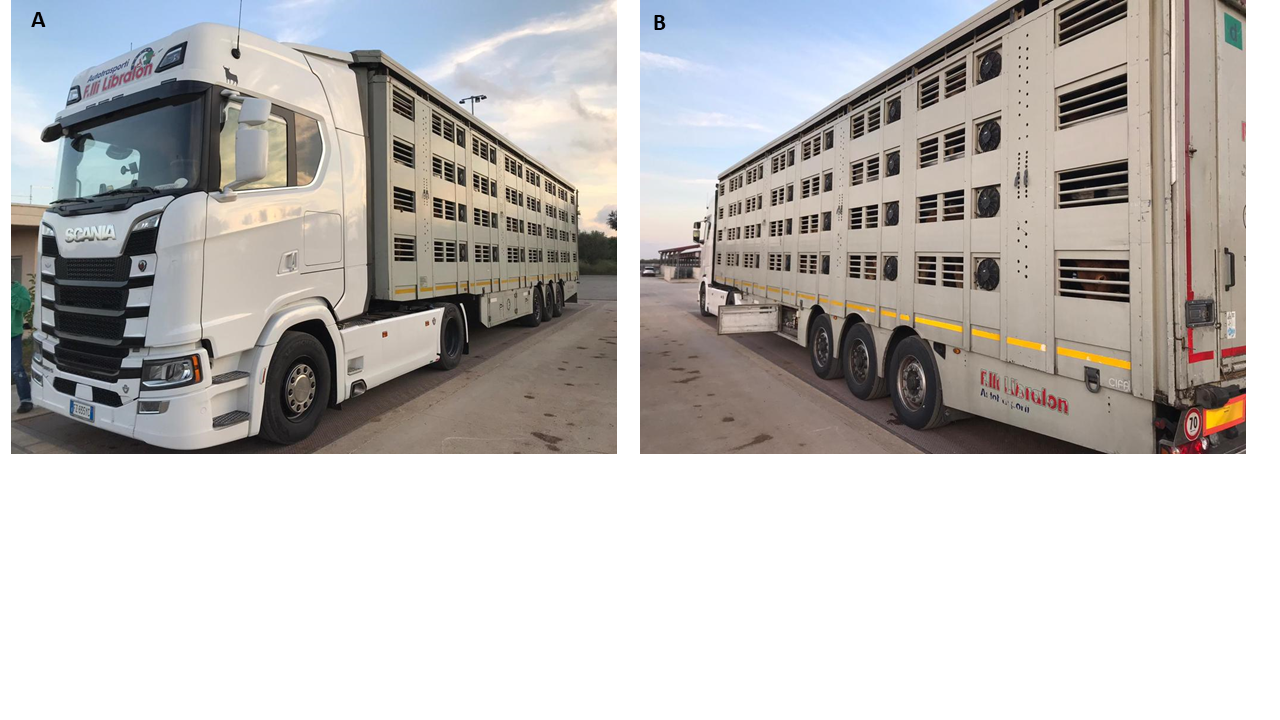


**Supplementary Figure 1.** Front (A) and rear (B) views of the vehicles used in the present field study on 169 beef steers transported from France to Italy. The vehicles were 4X2 cab over engine prime movers with a three-axle enclosed flatdeck semitrailer configured with two decks.


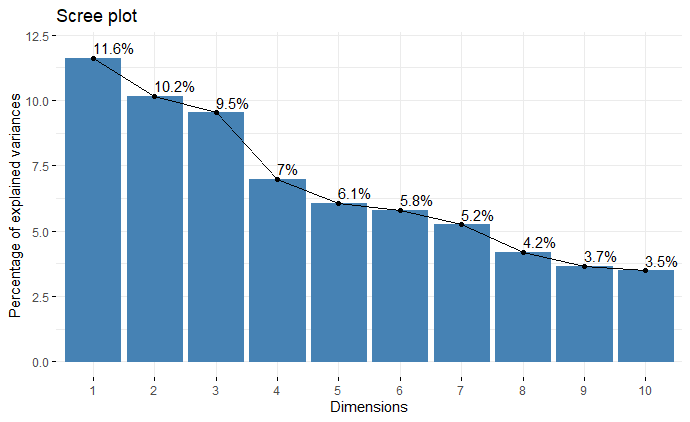


**Supplementary Figure 2.** The percentage of total inertia (or variance) explained by the first ten dimensions identified by the MCA.
